# Partial redundancy buffers deleterious effects of mutating *DNA methyltransferase 1-1* (*MET1-1*) in polyploid wheat

**DOI:** 10.1093/jxb/eraf016

**Published:** 2025-04-07

**Authors:** Samuel Burrows, Delfi Dorussen, Joseph Crudgington, Giorgia Di Santolo, James Simmonds, Marco Catoni, Philippa Borrill

**Affiliations:** Department of Crop Genetics, John Innes Centre, Norwich Research Park, Norwich NR4 7UH, UK; Department of Crop Genetics, John Innes Centre, Norwich Research Park, Norwich NR4 7UH, UK; Department of Crop Genetics, John Innes Centre, Norwich Research Park, Norwich NR4 7UH, UK; Department of Crop Genetics, John Innes Centre, Norwich Research Park, Norwich NR4 7UH, UK; Department of Crop Genetics, John Innes Centre, Norwich Research Park, Norwich NR4 7UH, UK; School of Biosciences, University of Birmingham, Birmingham B15 2TT, UK; Department of Crop Genetics, John Innes Centre, Norwich Research Park, Norwich NR4 7UH, UK; University Clermont Auvergne, France

**Keywords:** DNA methylation, DNA methyltransferases, epigenetics, gene dosage, polyploidy, wheat (*Triticum aestivum*)

## Abstract

DNA methylation plays important roles in gene expression, transposable element silencing, and genome stability. Altering DNA methylation could generate additional phenotypic variation for crop breeding, however the lethality of epigenetic mutants in crop species has hindered its investigation. Here, we exploit partial redundancy between homoeologues in polyploid wheat to generate viable mutants in the *DNA methyltransferase 1-1* (*MET1-1*) gene with altered methylation profiles. In *Triticum turgidum* (tetraploid wheat) and *Triticum aestivum* (hexaploid wheat), we found under-representation of higher order mutants (5/6 and 6/6 mutant *met1-1* copies in hexaploid wheat and 3/4 and 4/4 copies in tetraploid wheat) when genotyping segregating seeds and seedlings, due to reduced transmission of null mutant gametes from the paternal and maternal side. The loss of four or more functional copies of *MET1-1* results in decreased CG methylation in hexaploid wheat. Changes to gene expression increase stepwise with the number of mutant alleles, suggesting a dosage-dependent effect. We identified heritable changes to flowering and awn phenotypes which segregate independently of *MET1-1*. Together our results demonstrate that polyploidy can be leveraged to generate quantitative changes to CG methylation without the lethal consequences observed in other crops.

## Teaser

Varying gene dosage through mutating *DNA methyltransferase 1-1* (*MET1-1* ) in polyploid wheat generates quantitative changes to CG methylation without the lethal consequences observed in other crops.

## Introduction

DNA methylation on cytosine bases is an epigenetic modification which is conserved across biological kingdoms and plays important roles in gene expression, transposable element (TE) silencing, and genome stability. In plants, cytosine methylation can occur in any context (CG, CHG, and CHH, where H is A, T, or C), although CG methylation is the most prevalent context in plant species examined to date (Feng *et al.*, 2010; Niederhuth *et al.*, 2016; Domb *et al.*, 2020; Li *et al.*, 2023). Methylation is established and maintained by multiple enzymes which function in specific or overlapping contexts. CG methylation is maintained during cell division by methyltransferase 1 (MET1) which recognizes hemi-methylated CG sites following DNA replication and methylates the opposite strand (Kankel *et al.*, 2003; Zhang *et al.*, 2018).

Disruption of the *MET1* gene in Arabidopsis (*Arabidopsis thaliana*), tomato (*Solanum lycopersicum*), and rice (*Oryza sativa*) has revealed major differences in the phenotypic impact and severity of null mutations between species. Arabidopsis *met1* mutants, including complete and partial loss-of-function alleles, exhibit dwarfism, delayed flowering, abnormal embryo development, and seed abortion (Kankel *et al.*, 2003; Saze *et al.*, 2003; Xiao *et al.*, 2006; Srikant *et al.*, 2022). Null *met1* alleles have severe consequences, with segregation distortion resulting in an under-representation of homozygous mutants, which are only occasionally viable to maturity (Saze *et al.*, 2003; Srikant *et al.*, 2022). In rice, null homozygous mutants were formed at the expected ratio, but underwent necrotic death at the seedling stage (Hu *et al.*, 2014), and in tomato, homozygous null mutants could not be recovered (Yang *et al.*, 2019). These species also vary in CG methylation loss in *met1* mutants, with a stronger reduction in Arabidopsis than in rice (98.3% versus 75.7%) and only a 25% reduction in tomato CRISPR mutants, although this may be due to chimerism (Cokus *et al.*, 2008; Hu *et al.*, 2014; Yang *et al.*, 2019).

Differences in the segregation and viability of homozygous null mutants may be due to variation in epigenetic back-up systems: in Arabidopsis, these back-up systems operate through changes to the RNA-directed DNA methylation (RdDM) process, changes to expression of DNA demethylases, and progressive H3K9 remethylation of heterochromatin (Mathieu *et al.*, 2007). Stochasticity in these processes explains the rarity of homozygous null mutants (Mathieu *et al.*, 2007). In rice, a duplicate copy of *MET1* has been proposed to provide a back-up mechanism for essential CG methylation, although the lethality of homozygous mutants at the seedling stage indicates incomplete compensation (Hu *et al.*, 2014; Yamauchi *et al.*, 2014).

Mutants in *met1* have not been examined within a polyploid species where duplicate gene copies could provide a back-up mechanism. Alternatively, gene copies could be dosage sensitive, with a mutation in just one copy of *met1* affecting DNA methylation. The gene balance hypothesis proposes that dosage-sensitive genes are generally involved in macromolecular complexes or are regulatory genes such as those encoding transcription factors, rather than those encoding enzymes such as *MET1* (Veitia and Birchler, 2010; Birchler and Veitia, 2012). Moreover, it is generally expected that mutations in enzymes would be recessive, making it unlikely to find phenotypic effects in a partial *met1* mutant in a polyploid (Kondrashov and Koonin, 2004). However, some enzymes are dosage sensitive in polyploids, such as the E3 ubiquitin ligase gene *GW2* which has a dosage effect on grain width in wheat (Wang *et al.*, 2018). MET1 has been reported to interact at the protein level with histone deacetylase HDA6 and MEDEA in Arabidopsis (Liu *et al.*, 2012; Schmidt *et al.*, 2013), making a dosage effect more likely since it could be part of a macromolecular complex. Opposing this view, heterozygous mutants in tomato and rice maintained similar CG methylation to wild-type (WT) plants (Hu *et al.*, 2014; Yang *et al.*, 2019), indicating that only one allele was sufficient to compensate, and the same was found for some Arabidopsis accessions, although CG methylation was reduced up to 64% in others (Srikant *et al.*, 2022). Therefore, it is difficult to predict whether a partial mutant in *MET1* in a polyploid species would induce changes to CG methylation.

If *MET1* is dosage sensitive in polyploids, partial *met1* mutants could be used to alter methylation status and produce stable epialleles for use in plant breeding. Epialleles affecting diverse traits have been identified in a range of plant species, including alterations in floral morphology, fruit ripening, plant height, and climate adaptation (Cubas *et al.*, 1999; Manning *et al.*, 2006; Miura *et al.*, 2009; He *et al.*, 2018; Srikant and Tri Wibowo, 2021). The deliberate induction of stable epialleles has been demonstrated through epigenetic recombinant inbred line (epiRIL) populations generated genetically using *met1* and *ddm1* (*Decreased DNA Methylation 1*) mutants in Arabidopsis (Johannes *et al.*, 2009; Reinders *et al.*, 2009; Zhang *et al.*, 2013; Catoni and Cortijo, 2018). The potential to exploit novel epialleles in plant breeding has been proposed, but the production of epiRIL populations in crop species as a foundation to generate novel epialleles has been limited by the lethality of mutants (Springer and Schmitz, 2017). For example, no null mutants were identified in *MET1* orthologues in crop species with large genomes, including in a barley TILLING population (genome size of ~5 Gb) (Schreiber *et al.*, 2019) and in maize TILLING and transposon populations (genome size of ~2.4 Gb) (Li *et al.*, 2014). This may be due to the large numbers of TEs in large genomes causing strong genetic and developmental defects in *met1* mutants through their remobilization (Mirouze and Vitte, 2014).

Here we explore whether it is possible to use a polyploid crop species to generate quantitative changes to CG methylation by knocking out differing numbers of *met1* alleles, without causing lethal consequences. We use polyploid wheat as a model system due to the availability of sequenced mutant populations which can be readily applied to breeding worldwide without legislative complications surrounding gene editing (Krasileva *et al.*, 2017). We generate *met1* mutants in tetraploid (*Triticum turgidum*) and hexaploid (*Triticum aestivum*) wheat with up to three null alleles (of four) in tetraploid wheat, and five null alleles (of six) in hexaploid wheat. Contrary to the predictions of the gene dosage balance hypothesis (Veitia and Birchler, 2010), we find changes to global DNA methylation, transcriptional responses, and effects on fertility and plant growth linked to mutant allele copy number. We also identify stably inherited phenotypic alterations. Our findings provide insights into using polyploidy to generate quantitative changes to CG methylation for epigenetic breeding in crops.

## Materials and methods

### Protein sequence alignment

Amino acid sequences for the proteins encoded by *TraesCS2A02G235900* (*MET1-A1*), *TraesCS2B02G260800* (*MET1-B1*), and *TraesCS2D02G241800* (*MET1-D1*) were obtained from the EnsemblPlants database. Pairwise sequence alignments were made using the pwalign package in R (v4.4.1), from which the sequence identity was calculated.

### Plant materials and growth

**Fig. 1.**
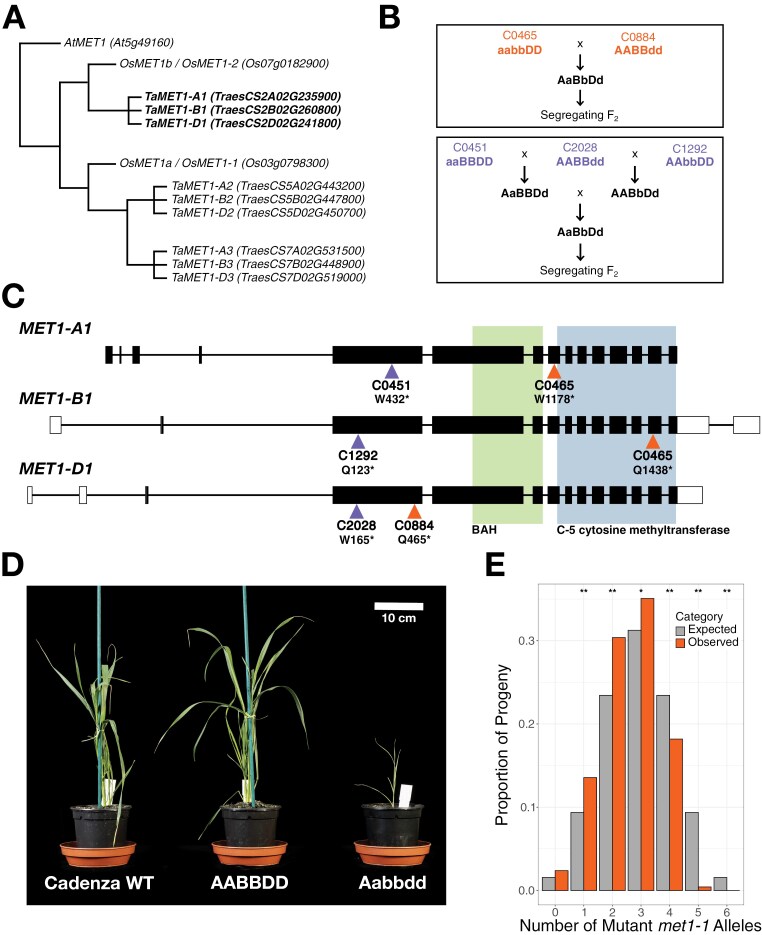
Null *met1-1* mutants cannot be produced by crossing TILLING lines with disruptions in the three *MET1-1* homoeologues. (A) Evolutionary relationships between *MET1* homologues in Arabidopsis (*AtMET1*), rice (*OsMET1a* and *OsMET1b*), and wheat (homoeologue groups *TaMET1-1*, *TaMET1-2*, and *TaMET1-3*). (B) Crossing schematic showing the generation of the hexaploid *MET1-1* populations: the C0465×C0884 population (top) and the C0451×C2028×C1292 population (bottom). (C) Structure of the three *MET1-1* homoeologues. Filled rectangles represent exons, open rectangles represent untranslated regions, and lines represent introns. Triangles indicate the positions of the premature termination codon mutations in the TILLING lines used to produce the two hexaploid F_2_ populations (C0465×C0884, orange; C0451×C2028×C1292, purple). The green box signifies the Bromo adjacent homology domain (BAH) and the blue box signifies the C-5 cytosine methyltransferase domain. (D) Cadenza WT, WT segregant (AABBDD), and Aabbdd individuals from the C0465×C0884 segregating F_2_ population. (E) Expected (grey) and observed (orange) proportion of progeny with each possible number of mutant *met1-1* alleles in the C0465×C0884 segregating F_2_ population, *n*=3608. Asterisks show adjusted *P*-values where the observed number of progeny differs significantly from the expected number; *P<0.05, **P<0.01.

Exome capture-sequenced ethyl methanesulfonate (EMS)-mutagenized populations of hexaploid (*Triticum aestivum* cv. Cadenza) and tetraploid wheat (*Triticum turgidum* cv. Kronos) were screened for premature termination codon mutations in *DNA methyltransferase 1-1* (*MET1-1*; *TraesCS2A02G235900*, *TraesCS2B02G260800*, and *TraesCS2D02G241800*) predicted to eliminate C5 cytosine methyltransferase domain activity (Fig. 1C) (Krasileva *et al.*, 2017).

Two independent Cadenza *MET1-1* populations were developed. The C0465×C0884 population was developed by crossing the double *met1-1* mutant Cadenza0465 with Cadenza0884 to generate a triple heterozygous F_1_, from which a segregating F_2_ population was produced (Fig. 1B). The C0451×C2028×C1292 population was developed by crossing Cadenza0451 with Cadenza2028 and Cadenza2028 with Cadenza1292. The F_1_ progeny were crossed to produce a triple heterozygous F_1_ which was self-pollinated to generate a segregating F_2_ population (Fig. 1B).

The Kronos *MET1-1* population was produced by crossing Kronos3085 with Kronos0809, giving double heterozygous F_1_ progeny, which were selfed to generate a segregating F_2_ population. From the F_2_ population, seeds from double heterozygous plants were sown to give a segregating F_3_ generation. F_4_ seeds from double heterozygous F_3_ plants were used for seed genotyping.

Seeds were germinated in Petri dishes at 4 °C for 48 h, followed by room temperature for 48 h before sowing into 96-cell trays containing John Innes F_2_ starter+Grit (90% peat, 10% grit, 4 kg m^–3^ dolomitic limestone, 1.2 kg m^–3^ osmocote start). Selected plants were potted on into 1 litre pots containing John Innes Cereal Mix (65% peat, 25% loam, 10% grit, 3 kg m^–3^ dolomitic limestone, 1.3 kg m^–3^ pg mix, 3 kg m^–3^ osmocote exact). Plants were grown in the John Innes Centre glasshouses (Norwich, UK) with supplementary lighting and heating as required for a minimum 16 h of light with 16 °C day and 14 °C night temperatures.

### Genotyping

#### Leaf

At the second leaf stage (Zadoks growth stage 12), DNA was extracted from 2 cm leaf samples following the protocol from https://www.wheat-training.com/ adapted from Pallotta *et al.* (2003). Genotyping was carried out using KASP (kompetitive allele-specific PCR) markers (Supplementary Table S1) and PACE mix, according to the manufacturer’s instructions. Kluster-Caller (v3.4.1.36) software was used for data analysis. We developed a cleaved amplified polymorphic sequence (CAPS) marker for C1292 (Supplementary Table S1), which was amplified using Taq DNA Polymerase (New England Biolabs, 94 °C for 2 min, 40 cycles of 94 °C for 15 s, 50 °C for 25 s, 72 °C for 1 min, ending with 2.5 min final extension at 72 °C). The product was digested by restriction enzyme *Pst*I-HF (New England Biolabs, 37 °C, 1 h). Homozygous *MET1-1* plants produced three digestion products (94, 157, and 249 bp), and homozygous *met1-1* mutants produced two digestion products (94 bp and 406 bp).

#### Grain

Grains were surface sterilized and cut transversely. The half of the grain containing the embryo was germinated and the other half (containing endosperm) was used for genotyping. Full details are available at https://www.protocols.io/view/genotyping-wheat-grains-to-identify-parental-donat-6qpvr84xplmk/v1. Parental donation of the *met1-1* allele was determined using KASP genotyping, where the usual single heterozygous cluster was split into two, corresponding to maternal or paternal donation of the mutant *met1-1* allele. This method was validated using reciprocal crosses between *Rht-B13b* (semi-dwarf) and WT plants employing KASP markers from Borrill *et al.* (2022).

### Phenotyping

#### Grain size measurements

Grains were graded into size categories using 3D printed sieves with apertures from 1.0 mm to 3.0 mm increasing by 0.5 mm increments The sieve design is available at https://www.printables.com/model/890045-grain-sieve. Following grain size grading, grain genotyping was carried out. The effect of the total number of mutant *met1-1* alleles and inheritance of an abd gamete on grain size was tested using an ordered logistic regression model with the MASS package in R (v4.2.2).

#### Pollen assays

Plants used for the pollen assays were grown in a randomized block design. Number of pollen grains per anther and pollen grain diameter were analysed using the Multisizer 4e Coulter Counter (Beckman Coulter, CA, USA). Per plant, three non-extruded, mature anthers were collected from three florets on the secondary spike. Anthers were stored in 70% ethanol at 4 °C. Pollen grains were released and analysed as previously described (Alabdullah *et al.*, 2021). Two measurements were performed per floret sample; one measuring all pollen grains in a total volume of 2 ml (to calculate mean pollen grains per anther) and another measuring up to 10 000 particles (to calculate modal pollen diameter). To remove the effect of debris, only particles with diameter between 30 µm and 60 µm were considered for this analysis.

Pollen viability was analysed using the Ampha Z32 Pollen Analyzer (Amphasys, Lucerne, Switzerland). Ten non-extruded, mature anthers were collected per plant from the primary spike and ruptured to release the pollen grains into AmphaFluid9 buffer (Amphasys). Up to 10 000 pollen grains were measured per sample; samples with <2000 pollen grains were excluded from the analysis. To identify the cluster corresponding to non-viable pollen grains, anthers from Cadenza WT plants were heated to 65 °C to kill the pollen. Separately, we agitated a sample of AmphaFluid9 buffer to identify the cluster corresponding to bubbles. Linear mixed models were used to calculate the estimated marginal means for pollen number per anther, pollen size, and percentage pollen viability, per genotype category, with block as a random variable [phenotype~genotype category+(1|block)]. Pairwise significant differences were assigned using a false discovery rate (FDR) multiple testing correction. Statistical analysis was carried out using the lme4 and emmeans packages in R (v4.2.2).

To determine the number of nuclei per pollen grain, pollen grains were stained with DAPI. Four non-extruded, mature anthers from the primary spike were fixed in Carnoy’s solution. After a minimum incubation of 48 h, the anthers were removed from the Carnoy’s solution and stained with DAPI solution [0.001 mg ml^–1^ DAPI, 1% (v/v) Triton X-100]. Pollen grains were released by sonication and incubated in the dark for 5 min. Samples were imaged with an Axio Zoom v16 II stereomicroscope (Zeiss, Oberkochen, Germany) with an ORCA-Flash4.0 Digital CMOS camera (Hamamatsu Photonics, Shizuoko, Japan). Six non-overlapping images were collected per sample. Non-, mono-, bi-, and tri-nucleated pollen grains were counted for each sample. Pollen grains with ambiguous numbers of nuclei were disregarded. As fewer replicates were used for DAPI staining, estimated marginal means were calculated irrespective of blocks, and significant differences were assigned by a Wilcoxon signed-rank test with FDR multiple testing correction. The percentages of pollen grains with abnormal numbers of nuclei were log transformed before the test was applied.

#### Flowering time

A late flowering plant from the C0451×C2028×C1292 F_2_ population was selfed. The progeny were genotyped and the date of complete emergence of the primary spike (Zadok’s growth stage Z59) recorded. Plant height and the length of the primary spike were measured. The number of fully formed spikelets on the primary spike was counted.

#### Awn development

An awned plant from the C0451×C2028×C1292 F_2_ population was crossed to Cadenza WT. BC_1_F_1_ plants were grown and selfed.

A total of 215 segregating BC_1_F_2_ plants were grown and scored for the presence of awns as previously described (Huang *et al.*, 2020). Fully awned individuals had awns >1 cm throughout the spike, whereas awnletted plants had awns <1 cm in the mid and basal portions of the spike while the length of awns at the apex reached up to 3 cm. We scored any plants that had awns <1 cm across the entire spike as awnless. We investigated the *Tipped1* (*B1*) gene (*TraesCS5A02G542800*), a characterized awn suppression gene, using published PCR and KASP markers (Huang *et al.*, 2020).

### Reciprocal crossing

To test the transmissibility of *met1-1* mutant alleles in each of the parental gametes, we made reciprocal crosses between Cadenza WT and triple heterozygous plants (AaBbDd) from the C0465×C0884 population. The seeds of these crosses were harvested and grown. Once the seedlings reached the two-leaf stage (Zadoks growth stage Z12), leaf genotyping was carried out.

### Whole-genome bisulfite sequencing

We collected leaf samples from the fourth leaf at Zadok’s growth stage Z15.6/23, or from the sixth leaf at Z16 for the Aabbdd mutant. Samples were frozen in liquid nitrogen and stored at –70 °C. DNA was extracted using the DNeasy Plant Mini Kit (Qiagen, Hilden, Germany) from three biological replicates per genotype, excluding the Aabbdd genotype, of which only one plant was recovered. DNA concentration was measured using an Invitrogen Qubit 4 fluorometer (Thermo Fisher Scientific, MA, USA) and the three replicates per genotype were pooled in equimolar ratios. Library preparation, bisulfite conversion, and sequencing by Illumina NovaSeq 6000 were performed by BMKgene (Münster, Germany), to obtain an average of 2.62 billion 150 bp paired-end reads per sample. Bisulfite conversion rates were calculated by BMKgene using the Bismark software (Krueger and Andrews, 2011), and rates between 99.52% and 99.70% were obtained. Fastp (Chen *et al.*, 2018) was used to remove adapter sequences, low quality reads, and poly(G) sequences. The Bismark Bisulfite Mapper (Krueger and Andrews, 2011) was used to process the sequencing data. Reads were aligned to a bisulfite-converted version of the IWGSC RefSeq v1.0 reference genome [International Wheat Genome Sequencing Consortium (IWGSC), 2018]. The average mapping efficiency was 40.49%, resulting in average coverage of 11×. Methylation calls in all sequence contexts (CG, CHG, and CHH) were extracted. Average cytosine methylation across genes and TEs was plotted using deepTools (Ramírez *et al.*, 2016) employing GTF files generated using gffread (Pertea and Pertea, 2020) from previously published gff3 files [International Wheat Genome Sequencing Consortium (IWGSC), 2018; Wicker *et al.*, 2018]. Only cytosines with at least four reads were included in the analysis. Methylation profiles across chromosomes were calculated using computeMethylationProfile from DMRCaller (Catoni *et al.*, 2018), with bins of 1 Mb. Partitions between chromosome segments (R1, R2a, C, R2b, and R3) were assigned based on the segments defined in International Wheat Genome Sequencing Consortium (IWGSC) (2018) available at https://opendata.earlham.ac.uk/wheat/under_license/toronto/Ramirez-Gonzalez_etal_2018-06025-Transcriptome-Landscape/data/TablesForExploration/. Differentially methylated regions (DMRs) in the CG context were called for each chromosome separately using computeDMRs from DMRCaller (Catoni *et al.*, 2018), specifying the method as ‘bins’, binSize as 100 bp, pValueThreshold as 0.01, minCytosinesCount as 10, minProportionDifference as 0.4, minGap as 0, and minReadsPerCytosine as 4. DMRs associated with genomic features were identified using bedtools intersect (Quinlan and Hall, 2010) to find overlapping genomic coordinates between previously annotated high-confidence genes (including 1 kb up- and downstream of the coding sequence) [International Wheat Genome Sequencing Consortium (IWGSC), 2018] and TEs (Wicker *et al.*, 2018).

### RNA-sequencing

We collected leaf samples from the fourth leaf at Zadok’s growth stage Z15.6/23, or from the sixth leaf at Z16 for the Aabbdd mutant. The samples were frozen in liquid nitrogen and stored at –70 °C. RNA was extracted using Trizol–chloroform and the RNA Clean & Concentrator Kit (Zymo Research). RNA was extracted from three biological replicates per genotype, excluding the Aabbdd genotype. Library preparation and sequencing by Illumina NovaSeq 6000 was performed by BMKgene (Münster, Germany), to obtain 150 bp paired-end reads. Fastp (Chen *et al.*, 2018) was used to remove adapter sequences, low quality reads, and poly(G) sequences. Reads were pseudoaligned to the IWGSC RefSeq v1.1 reference transcriptome [International Wheat Genome Sequencing Consortium (IWGSC), 2018] and quantified using kallisto (Bray *et al.*, 2016). The output from kallisto was imported into R (v4.3.2) using tximport (Soneson *et al.*, 2015). Differentially expressed genes (DEGs) between genotypes were identified using DESeq2, utilizing an adjusted *P*-value threshold of <0.01 and ≥2-fold change (Love *et al.*, 2014). For TE expression, reads were mapped to the IWGSC reference sequence using HISAT2 (Pertea *et al.*, 2016), and reads uniquely mapping to complete TE positions (Wicker *et al.*, 2018) were counted using HTSeq (Anders *et al.*, 2015), with default parameters. Differential expression analysis was carried out as for genes.

Gene expression data across tissues was downloaded as tpm values from https://bar.utoronto.ca/efp_wheat/cgi-bin/efpWeb.cgi (Borrill *et al.*, 2016; Ramírez-González *et al.*, 2018).

## Results

### Null *met1-1* mutants cannot be recovered in polyploid wheat

*METHYLTRANSFERASE 1* (*MET1*) genes were previously identified in hexaploid wheat (*T. aestivum*) on the group 2, 5, and 7 chromosomes, with the group 5 and 7 *MET1* homologues predicted to be pseudogenes (Fig. 1A) (Thomas *et al.*, 2014). The group 2 genes are orthologous to *MET1b* in rice, also known as *OsMET1-2*, which strongly affects CG methylation (Fig. 1A) (Hu *et al.*, 2014). In an RNA-seq time course for the Azhurnaya variety, the group 2 genes (*TraesCS2A02G235900*, *TraesCS2B02G260800*, and *TraesCS2D02G241800*) are expressed in numerous above-ground tissues, whereas expression of the group 5 and group 7 genes was virtually undetectable in most of these tissues (Supplementary Fig. S1) (Ramírez-González *et al.*, 2018). We therefore concentrated our analysis on the group 2 genes which will be referred to as *MET1-A1*, *MET1-B1*, and *MET1-D1* in accordance with the gene nomenclature guidelines for wheat (Boden *et al.*, 2023) and a previous study by Li *et al.* (2021). The *MET1-1* homoeologues have a high degree of protein sequence identity, with MET1-B1 and MET1-D1 having the highest sequence identity (98.8%), followed by MET1-A1 and MET1-D1 (93.3%) and MET1-A1 and MET1-B1 (93.1%).

We produced independent segregating F_2_ populations in hexaploid wheat from which, in theory, all combinations of loss-of-function *met1-1* mutants could be recovered (Fig. 1B, C). However, we were unable to recover null *met1-1* mutants with homozygous loss-of-function mutations in all three homoeologues from either population, despite screening 3068 individuals from the C0465×C0884 population and 328 individuals from the C0451×C2028×C1292 population. Moreover, we only found one plant with five non-functional *MET1-1* alleles which was capable of germination, although its growth was severely stunted and the plant died before progressing to tillering (Zadoks growth stage 20) (Fig. 1D). The genotype ratios observed in both populations were significantly different from expected Mendelian ratios as individuals with four or more non-functional *MET1-1* alleles were under-represented (C0465×C0884 Fig. 1E, χ^2^*P*=1.63×10^–103^; C0451×C2028×C2192 Supplementary Table S2, χ^2^*P*=5.05×10^–12^). We concluded that higher order *met1-1* mutants have reduced viability in hexaploid wheat.

### Reduced viability of null *met1-1* gametes hinders recovery of higher order *met1-1* mutants

**Fig. 2.**
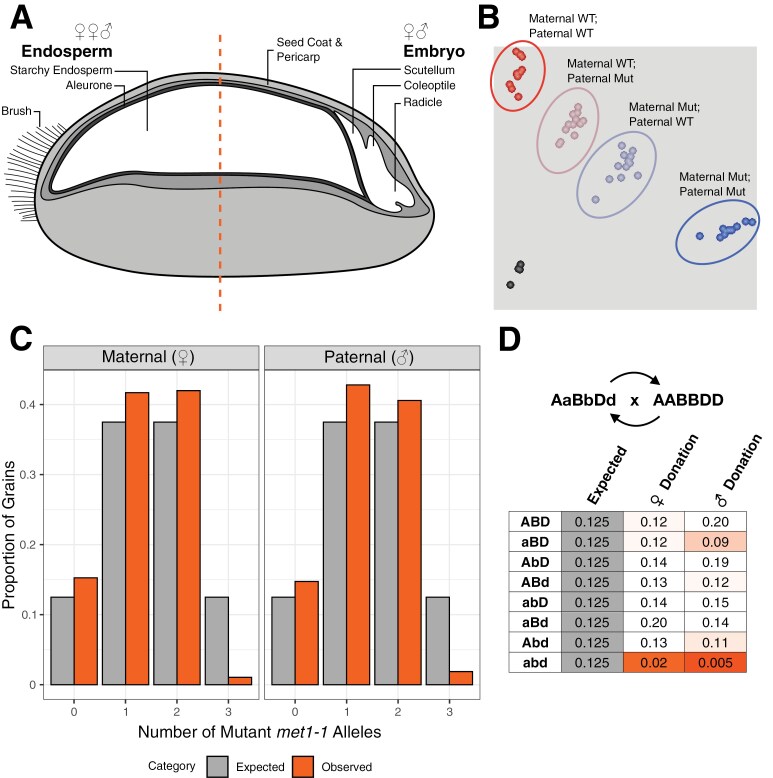
Inheritance of three mutant *met1-1* alleles from the female or male parent (i.e. abd gamete) is rare relative to inheritance of zero, one, or two mutant alleles. (A) Wheat grains divided transversely to separate two halves of the grain, with one half containing the embryo for future germination and the other half containing largely endosperm for DNA extraction and genotyping. (B) KASP genotyping for *Rht13* mutants (*Rht-B13b*) from reciprocal crosses between mutant and wild-type parents with known genotypes, showing homozygous wild-type (WT) (red cluster), homozygous mutant (blue cluster), heterozygous clusters with a maternally inherited WT allele and paternally inherited mutant allele (brown), a paternally inherited WT allele and maternally inherited mutant allele (grey), and no template control (black). (C) Expected (grey) and observed (orange) proportion of grains inheriting zero, one, two, or three mutant *met1-1* alleles from the maternal or paternal gamete from a self-pollinated AaBbDd plant, *n*=1710. (D) Expected and observed proportion of individuals formed from each type of gamete when inherited maternally (♀ donation) or paternally (♂ donation) from an AaBbDd parent when crossed with AABBDD. Orange shades indicate proportions below the expected proportion of 0.125.

We hypothesized that the absence of functional *MET1-1* alleles within a gamete might cause this segregation distortion. We developed a grain genotyping method using the endosperm-containing half of the grain (Fig. 2A) which carries maternal to paternal DNA at a 2:1 ratio (endosperm originates from two female central cell nuclei being fertilized by one male sperm cell). This allowed us to determine which parent donated mutant alleles to the offspring (grain) by separating the heterozygous genotypes into two clusters (Fig. 2B). We confirmed the accuracy of this method using reciprocal crosses for an independent mutation in the semi-dwarfing gene *Rht13* (Borrill *et al.*, 2022) (Fig. 2B). Grain genotypes were consistent with seedlings grown from the embryo-containing halves of the grain (Supplementary Table S3). We genotyped 1710 grains from a segregating F_2_ hexaploid wheat population (C0465×C0884) and observed far fewer grains which inherited three mutant *met1-1* alleles from one parent than expected (generalized linear model with log link, *P*<2×10^–16^; Fig. 2C). We detected fewer grains which inherited three mutant alleles from the maternal side than from the paternal side (18 versus 32 grains). There was no reduction in the number of grains which inherited one or two mutant alleles from the same parent (Fig. 2C).

We confirmed these observations in an independent segregating F_4_ tetraploid wheat population (332 grains) (Supplementary Fig. S2). We observed a significant segregation distortion (χ^2^*P*=3.39×10^–30^) and were unable to identify any homozygous null mutants (aabb). Tetraploid wheat gametes with a complete knockout of *MET1-1* (i.e. ab gametes) were also strongly under-represented (generalized linear model with log link, *P*<2×10^–16^), with reduced maternal and paternal transmission (Supplementary Table S4). In tetraploid wheat, we also detected fewer grains which inherited two mutant alleles from the maternal side than from the paternal side (1 versus 18 grains; Supplementary Table S4).

In hexaploid wheat, we found that the distribution of genotypes in grains was significantly different from that in seedling leaf tissue (χ^2^*P*=0.00058), with a greater proportion of individuals with five non-functional *MET1-1* alleles observed in the grain population [12 out of 1710 in the grain population (0.702%) compared with 1 out of 1352 in the seedling population (0.074%)]. This suggests that higher order *met1-1* mutants may be formed as grains, but not be capable of germination. We hypothesized that *met1-1* mutants inheriting three non-functional alleles from the same parent may be associated with decreased grain size. We found that for grains with a total of three or four mutant *met1-1* alleles, those inheriting three mutant alleles from one parent had a 39–46% decrease in grain width compared with those inheriting the mutant alleles separately from both parents (ordered logistic regression model, *P*<0.05; Supplementary Fig. S3).

To further understand why the recovery of *met1-1* mutants formed from at least one null mutant gamete is reduced, we investigated whether pollen development was affected in the *met1-1* mutants. We measured the number of pollen grains per anther, pollen diameter, pollen viability, and the proportion of pollen grains that were non- or mononucleated for plants from the segregating F_2_ population, including those that should be capable of producing gametes with no functional copies of *MET1-1* (AaBbdd, AabbDd, and aaBbDd). However, no significant differences (at α=0.05) were detected between those with an AaBbdd, AabbDd, or aaBbDd genotype and the WT segregant (AABBDD) for any of these phenotypes (Supplementary Table S5).

To confirm whether inheritance of three mutant *met1-1* alleles from the same parent reduces viability, we performed reciprocal crosses between triple heterozygous (AaBbDd) *met1-1* mutants and Cadenza WT. In total, 454 progeny were genotyped, 235 with AaBbDd as the female parent and 219 with AaBbDd as the male parent. Again, we found that plants inheriting three non-functional *MET1-1* alleles from one parent were significantly under-represented compared with the expected Mendelian ratio (χ^2^*P*<0.001 for both maternal and paternal donation; Fig. 2D). The proportion of abd gametes was lower from the paternal side of the reciprocal crosses than from the maternal side, opposite to the trend observed in the selfed progeny of a triple heterozygous plant (Fig. 2C).

### Loss of four or more functional copies of *MET1-1* results in decreased CG methylation

**Fig. 3.**
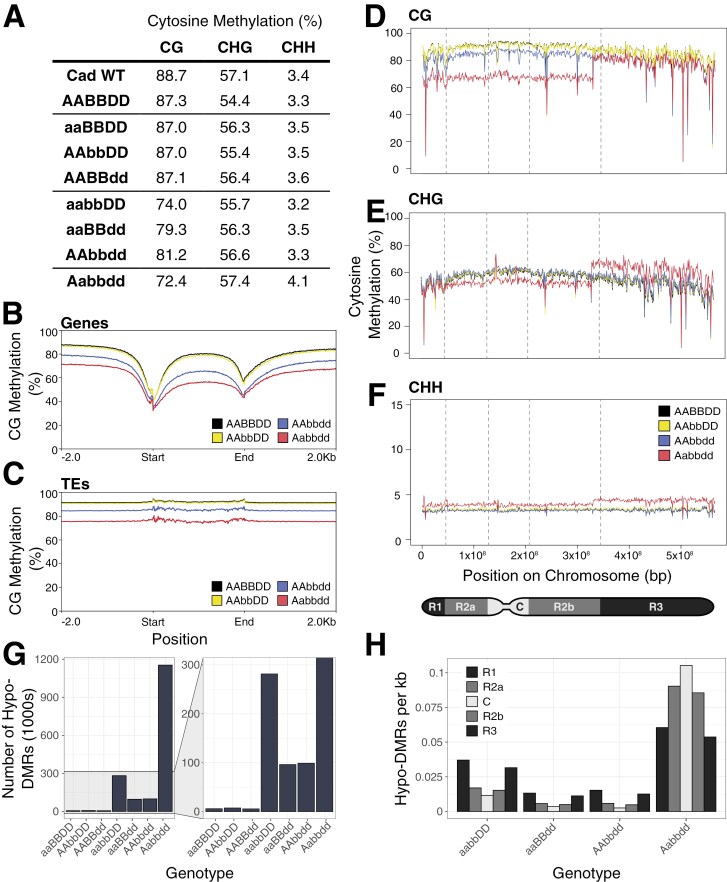
Reduced number of functional copies of *MET1-1* results in loss of CG methylation and the formation of differentially methylated regions (DMRs). (A) Genome-wide percentage of cytosines methylated in the CG, CHG, and CHH contexts. (B and C) Percentage of CG methylation across genes (B) and transposable elements (TEs) (C), including 2 kb up- and downstream of the feature. The WT segregant (AABBDD, black), AAbbDD single mutant (yellow), AAbbdd double mutant (blue), and Aabbdd mutant (red) are shown. (D–F) Percentage of methylated cytosines in the CG (D), CHG (E), and CHH (F) contexts across chromosome 5D, calculated as an average in 1 Mb bins across the chromosome. The WT segregant (AABBDD, black), AAbbDD single mutant (yellow), AAbbdd double mutant (blue), and Aabbdd mutant (red) are shown. Partitions between the chromosome regions are indicated by the dashed lines. (G) Number of hypomethylated differentially methylated regions (hypo-DMRs) in 1000s relative to AABBDD in each genotype. (H) Number of hypomethylated DMRs per kb in each of the chromosome regions—the distal regions R1 and R3 are shown in dark grey, the proximal regions R2a and R2b in medium grey, and the centromeric region C in light grey.

We carried out whole-genome bisulfite sequencing to determine whether a stepwise reduction in the number of functional *MET1-1* alleles was associated with a proportional change in DNA methylation. We observed partial functional redundancy between the *MET1-1* homoeologues; genome-wide CG methylation in the single mutants (aaBBDD, AAbbDD, and AABBdd) was between 87.0% and 87.1% which was very similar to the WT segregant (AABBDD, 87.3% CG methylation). Genome-wide CG methylation was reduced in the double mutants, ranging from 74.0% in the aabbDD mutant, to 79.3% in the aaBBdd mutant, and 81.2% in the AAbbdd mutant. The lowest level of CG methylation was observed in the Aabbdd mutant, with 72.4% (Fig. 3A). However, the Aabbdd mutant had the highest CHG (57.4%) and CHH (4.1%) methylation of all the lines tested (Fig. 3A).

To explore the impact of these global changes in methylation, we examined methylation across genes and TEs. Average CG methylation was decreased across genes and TEs relative to the WT segregant (AABBDD) in both the double mutant (AAbbdd) and the Aabbdd mutant (Fig. 3B, C). For both sequence features, the greatest decrease was observed for the Aabbdd mutant, in line with the global changes in methylation. No obvious difference was observed in CHG or CHH methylation across genes or TEs (Supplementary Fig. S4).

Spatial correlations between different methylation contexts were observed when looking at the distribution of cytosine methylation across each chromosome. For example, along chromosome 5D, CG methylation is reduced in the Aabbdd mutant compared with the WT segregant, and single and double mutants in the R1, R2a, C, and R2b regions. In the R3 region, however, CG methylation in the Aabbdd mutant is comparable with that in the AAbbdd double mutant (Fig. 3D). In this region, there is also an increase in CHG methylation in the Aabbdd mutant, such that it is higher than any of the other lines (Fig. 3E), and an increase in CHH methylation (Fig. 3F). There is a clear transition point between states in the Aabbdd mutant across all three methylation contexts (Supplementary Fig. S5). The simultaneous increase in CG and CHG/CHH methylation is also observed in specific regions of another 10 chromosomes, affecting all possible chromosome regions not only R3, and across the whole of chromosomes 3A, 4D, and 6B (Supplementary Figs S6–S12). These region-specific increases in CG, CHG, and CHH methylation were only observed in the Aabbdd mutant, and not in any of the single or double mutants, as shown for chromosome 5D (Supplementary Fig. S13).

To further understand the spatial nature of methylation changes in the *met1-1* mutants, we identified 100 bp DMRs in the CG context in each of the mutants relative to the WT segregant. Substantially more hypomethylated DMRs (hypo-DMRs) were detected in the Aabbdd mutant (1 153 473 hypo-DMRs), compared with between 96 406 and 281 591 hypo-DMRs in the double mutants, and between 5699 and 7565 hypo-DMRs in the single mutants (Fig. 3G). The vast majority of DMRs were hypomethylated in the *met1-1* mutants—only 0.27% of the DMRs in the Aabbdd mutant were hypermethylated (Supplementary Fig. S14). We observed that, in the double mutants, the distal regions R1 and R3 have the greatest density of hypo-DMRs, while the centromeric region C has the lowest density (Fig. 3H). However, in the Aabbdd mutant, the centromeric region has the highest density of hypo-DMRs (Fig. 3H). In all *met1-1* mutants, the density of hypermethylated DMRs is lowest in the proximal and centromeric regions, and highest in the distal regions (Supplementary Fig. S15).

### Reduced function of *MET1-1* results in stepwise changes in gene expression

**Fig. 4.**
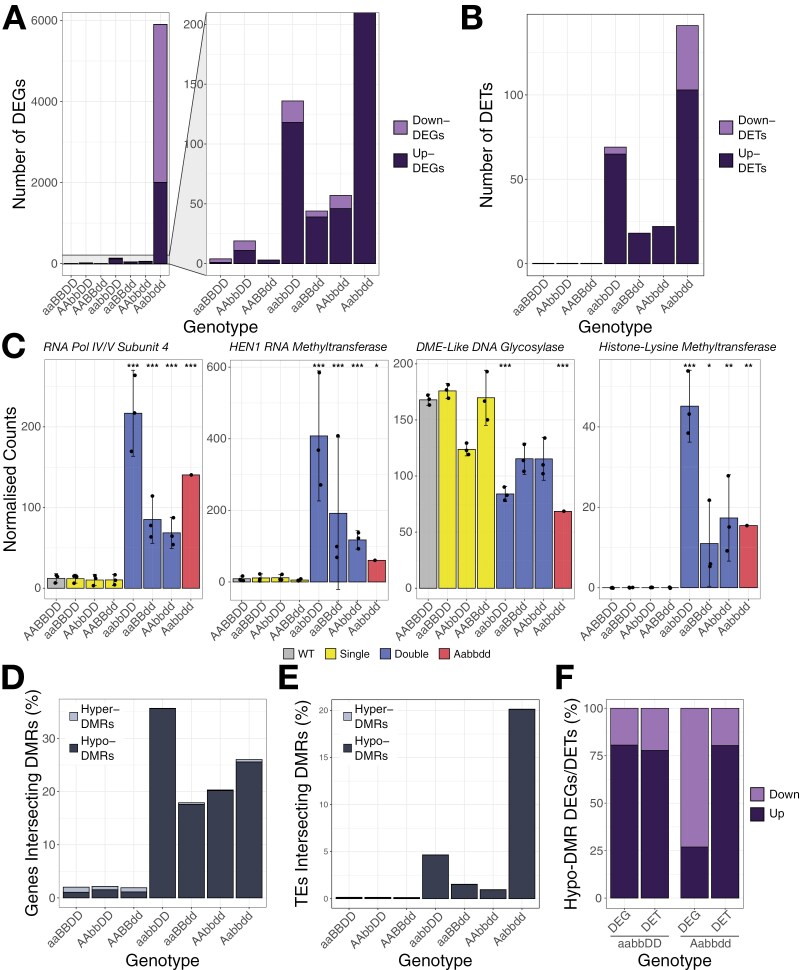
Reduced number of functional copies of *MET1-1* results in transcriptional reprogramming. (A) Number of differentially expressed genes (DEGs) relative to AABBDD in each genotype. Both down-regulated (light purple) and up-regulated (dark purple) genes are shown. (B) Number of differentially expressed transposable elements (DETs) relative to AABBDD in each genotype (uniquely mapped reads only). Both down-regulated (light purple) and up-regulated (dark purple) transposable elements are shown. (C) Normalized transcript counts in each genotype for genes putatively encoding an RNA Polymerase IV/V subunit (*TraesCS6B02G315200*), a HEN1 small RNA 2’-*O*-methyltransferase (*TraesCS2D02G114600*), a DEMETER (DME)-like DNA glycosylase (*TraesCS3B02G023200*), and a histone-lysine methyltransferase (*TraesCS3B02G503200*). Error bars represent the 95% confidence interval, estimated as 1.96×SE. Asterisks show adjusted *P*-values for differential expression relative to AABBDD; *P<0.05, **P<0.01, ***P<0.001. (D and E) The percentage of genes (D) or transposable elements (E) which intersect at least one DMR in each of the *met1-1* mutants. Hyper-DMRs are shown in light grey and hypo-DMRs are shown in dark grey. (F) The percentage of DEGs or DETs associated with a hypo-DMR which are down-regulated (light purple) or up-regulated (dark purple) in the aabbDD and Aabbdd *met1-1* mutants.

To determine the effect of these changes in methylation, we conducted RNA-sequencing and differential expression analysis. The number of DEGs compared with the WT segregant (FDR adjusted *P*<0.01, fold change ≥2) was calculated for each mutant class (Fig. 4A). The Aabbdd mutant had the largest number of DEGs (5904), while double mutants had on average 79 DEGs, and single mutants had on average nine DEGs. Generally, there were more up-regulated DEGs than down-regulated DEGs, except for the Aabbdd mutant, with 3900 genes down-regulated and 2004 genes up-regulated (Fig. 4A). There was also a stronger change in TE expression in the double and Aabbdd mutants compared with single mutants, in which no differentially expressed TEs (DETs) were observed (on average 36 DETs in double mutants and 143 DETs in the Aabbdd mutant; FDR adjusted *P*<0.01, fold change ≥2, Fig. 4B). These changes in gene and TE expression are in line with the global changes in CG methylation, with mutants maintaining the highest level of CG methylation relative to the WT segregant having the fewest DEGs, and vice versa (Figs 3A, 4A, B).

To further examine the transcriptional response, we identified genes that were differentially expressed in both the Aabbdd mutant and at least one of the double mutants. A total of 86 DEGs were identified in this way, 76 of which were up-regulated and 10 of which were down-regulated. Several of these DEGs have putative functions relating to DNA methylation and other epigenetic marks (Fig. 4C), suggesting that reduced *MET1-1* function initiates active changes in transcriptional regulation. The gene *TraesCS6B02G315200* is up-regulated in all double mutants and the Aabbdd mutant (adjusted *P*<0.01) (Fig. 4C), and is annotated in the Panther Classification System as RNA Polymerase IV and V Subunit 4 (PTHR21297:SF2). The homoeologous gene *TraesCS6D02G266900* is also up-regulated in all double mutants and the Aabbdd mutant, while its other homoeologue *TraesCS6A02G286200* and tandem duplicated gene copy *TraesCS6B02G15300* are up-regulated only in the aabbDD and Aabbdd mutants. A gene with the same Panther classification in Arabidopsis, *NRPD4* (*AT4G15950*), is required for RdDM (He *et al.*, 2009). The gene *TraesCS2D02G114600* was also up-regulated in all double *met1-1* mutants (Fig. 4C). This gene encodes a small RNA 2’-*O*-methyltransferase annotated as HEN1 in the Panther Classification System (PTHR21404). In Arabidopsis, *HEN1* is also implicated in RdDM (Erdmann and Picard, 2020). Furthermore, in both the aabbDD and Aabbdd mutants, *TraesCS3B02G023200*, encoding a DEMETER (DME)-like DNA glycosylase, is significantly down-regulated (Fig. 4C). In Arabidopsis, loss-of-function mutations in DME-like DNA glycosylase genes *DML2* and *DML3* result in increased cytosine methylation in regions with low to intermediate methylation levels in the WT (Ortega-Galisteo *et al.*, 2008). Overall, this suggests that reduced *MET1-1* function is buffered by up-regulation of genes that facilitate *de novo* DNA methylation and down-regulation of genes that remove DNA methylation.

We also observed up-regulation of *TraesCS2B02G503200* in the Aabbdd, aabbDD, and AAbbdd mutants (adjusted *P*<0.01), and in the aaBBdd mutants (adjusted *P*<0.05) (Fig. 4C). This gene is predicted to contain a histone-lysine *N*-methyltransferase SUVR4/SUVR1/SUVR2 domain (IPR025776). Reduced function of *MET1-1* may therefore promote changes in histone methylation that could further alter transcription.

Next, we investigated whether the CG-DMRs identified in the *met1-1* mutants (Fig. 3) overlapped genes and TEs. Up to 35.7% of high-confidence genes International Wheat Genome Sequencing Consortium (IWGSC), 2018] overlapped with CG-DMRs in the *met1-1* mutants (either within the gene body, or within 1 kb up- or downstream) (Fig. 4D). Although the Aabbdd mutant had 4.1–11.7 times as many CG-DMRs as the double mutants (Fig. 3G), the percentage of genes intersected by at least one CG-DMR (26.0%) was within the range observed in the double mutants (between 17.9% and 35.7%). In line with the overall distribution of DMRs, the majority of DMRs intersecting genes were hypomethylated in the *met1-1* mutants (Fig. 4D).

In contrast to the DMRs intersecting genes, we observed a stark difference between the percentage of TEs overlapped by at least one CG-DMR in the Aabbdd mutant and the double mutants (Fig. 4E). In the Aabbdd mutant, 20.1% of non-fragmented TEs (Wicker *et al.*, 2018) were intersected by at least one DMR, compared with between 0.95% and 4.7% in the double mutants. This is in line with the greater density of hypo-DMRs in the transposon-dense centromeric region in the Aabbdd mutant (Fig. 3H). A total of 87.0% of hypomethylated TEs in the Aabbdd mutant were long terminal repeat retrotransposons (LTR-RTs), compared with between 56.5% and 72.5% in the double mutants (Supplementary Table S6).

To examine whether changes in methylation status may be directly related to changes in gene expression, we quantified the percentage of DEGs which overlapped at least one CG-DMR. We focused on the double and Aabbdd mutants, as only a small number of DEGs were identified in the single mutants (Fig. 4A). Between 32.6% and 51.5% of DEGs were associated with a DMR. For each mutant, the proportion of DEGs associated with a DMR is greater than the proportion of genes associated with a DMR, suggesting that changes in DNA methylation may be directly responsible for the change in expression of a subset of DEGs. In the aabbDD double mutant, 80.6% of hypomethylated DEGs increased in expression relative to the WT segregant (Fig. 4F). However, in the Aabbdd mutant, only 26.9% of hypomethylated DEGs were up-regulated (Fig. 4F). This could reflect the overall greater proportion of down-regulated genes in the Aabbdd mutant compared with the double mutants (Fig. 4A). The majority of DETs associated with a hypo-DMR were up-regulated in both the aabbDD and Aabbdd mutants (77.8% in aabbDD and 80.4% in Aabbdd) (Fig. 4F). The same trend was observed for the other double mutants (Supplementary Fig. S16).

### Heritable phenotypic changes were identified in the *MET1-1* segregating F_2_ population

**Fig. 5.**
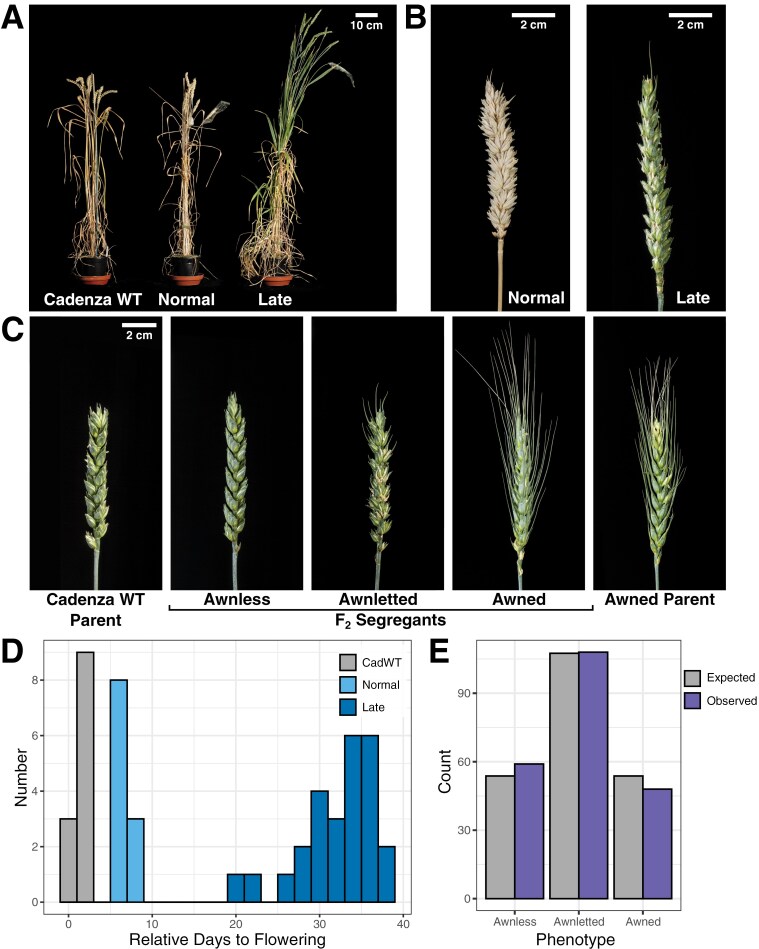
Heritable phenotypic changes in flowering time and presence of awns were observed in the C0451×C1292×C2028 *MET1-1* population. Late flowering and awned individuals were used to develop two separate segregating populations. (A) Phenotypes of Cadenza WT and normal and late flowering F_3_ segregant plants. (B) Spikes from normal and late flowering F_3_ segregant plants; the late flowering plant has an increased number of fully formed spikelets. (C) Phenotypes of the awnless Cadenza WT parent and a spontaneous awned individual which were crossed together to generate the F_1_ generation. The F_1_ was self-pollinated to generate the segregating F_2_ population which displayed awnless, awnletted, and awned phenotypes. (D) Histogram showing the days to flowering (full emergence of the primary spike) relative to the first plant, for the different flowering time categories in the segregating F_3_. (E) Expected (grey) and observed (purple) number of individuals which were awnless, awnletted, and awned in the segregating F_2_ population.

When growing the C0451×C1292×C2028 F_2_ population, we observed novel phenotypes in two plants, late flowering (Fig. 5A, B) and the presence of awns (Fig. 5C). The plants with these novel phenotypes had four non-functional *MET1-1* alleles (the late flowering individual was AaBbdd; the awned individual was AAbbdd). The late flowering plant flowered 23 d later than any other plant and had lax ears (thin ears composed of many spikelets). Offspring from this late flowering individual segregated into normal flowering types with normal ears (<10 d relative to the earliest flowering plant) and late flowering types with lax ears (≥10 d relative to the earliest flowering plant) (Fig. 5A, B, D). The numbers of segregant plants in the late and normal flowering categories did not significantly differ from a 3:1 ratio (χ^2^=0.214, *P*-value=0.644), suggesting that a single dominant gene controls the phenotype. The late flowering phenotype was independent of the *MET1-1* genotype (Supplementary Fig. S17A). The late flowering plants were also significantly taller than normal flowering plants (FDR adjusted *P*-value=8.2×10^–15^; Supplementary Fig. S17B). There was no significant difference in spike length between the late and normal flowering segregants (FDR adjusted *P*-value=0.22, Supplementary Fig. S17C); however, late flowering segregants had a higher number of fully formed spikelets per spike than normal flowering segregants (mean 25.3 versus 18.6; FDR adjusted *P*-value=5.5×10^–9^, Fig. 5B; Supplementary Fig. S17D). These data show that the tall, late flowering phenotype is heritable and is independent of *MET1-1* genotype, suggesting that it is controlled by a single stable allele generated in the mutant background.

The awned plant we found in the same segregating F_2_ population (C0451×C1292×C2028) was unexpected considering that all three parents were awnless. We generated a segregating population by backcrossing the awned plant with Cadenza WT and selfing the F_1_ generation, which showed an intermediate (awnletted) phenotype. Amongst 215 F_2_ plants, the segregation ratio of awnless:awnletted:awned individuals did not significantly deviate from a single co-dominant gene 1:2:1 Mendelian ratio (χ^2^=1.13, *P*-value=0.568) (Fig. 5C, E). The awnletted individual had longer awns at the top of the ear, similar to the effect of the *Tipped1* (*B1*) awn suppressor gene (Huang *et al.*, 2020), and we hypothesized that this was the causal gene controlling the phenotype. We used published primer sequences to screen the *B1* region in the F_2_ population (Huang *et al.*, 2020). PCR products could be obtained for awnless and awnletted plants, but not for awned plants either across the gene body or in the surrounding 28.5 Mbp (Supplementary Fig. S18). Together, these results indicate that the presence of awns in our population is due to a heritable deletion >28.5 Mbp which includes *B1*.

## Discussion

### Complete loss of *MET1-1* is lethal in wheat, but single and double mutants can be recovered

Despite genotyping >3000 progeny from two segregating *MET1-1* populations, we were unable to recover any null *met1-1* mutants in hexaploid wheat (Fig. 1F; Supplementary Table S2). Similarly, we were unable to recover any null *met1-1* mutants in tetraploid wheat (Supplementary Table S4). This indicates that complete loss of *MET1-1* is lethal in wheat, consistent with other monocotyledonous crop species with large genomes such as maize and barley (Li *et al.*, 2014; Schreiber *et al.*, 2019).

**Fig. 6.**
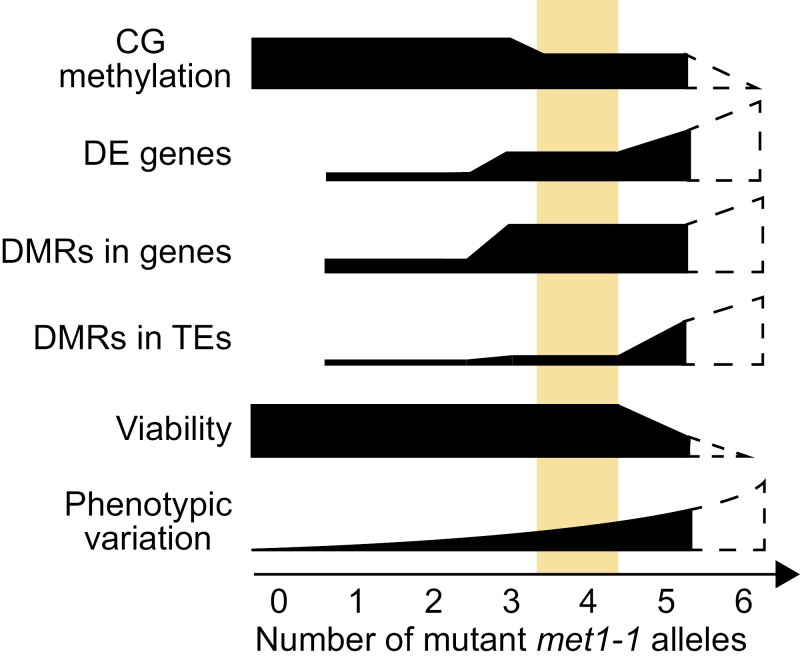
Effects of increasing the number of mutant *met1-1* alleles in hexaploid wheat. Increasing the number of mutant *met1-1* alleles has quantitative yet variable effects on CG methylation, differentially expressed (DE) genes, differentially methylated regions (DMRs) in genes and in transposable elements (TEs), viability, and phenotypic variation. Dashed regions indicate hypothetical data for a complete null mutant. The region highlighted in yellow represents an optimum where CG methylation is substantially reduced and viability is retained, made possible by partial redundancy between homoeologues.

However, unlike maize and barley, wheat is a polyploid, allowing intermediate mutants to be recovered. We recovered all possible homozygous single and double *met1-1* mutants from the segregating *MET1-1* populations, suggesting that the function of *MET1-1* homoeologues in wheat is mostly redundant. We also recovered a single Aabbdd mutant, in which five out of six copies of *MET1-1* are non-functional (Fig. 1D). We found that *MET1-1* has a threshold for dosage sensitivity: while the single mutants had no decrease in CG methylation compared with the WT segregant, the double and Aabbdd mutants showed altered patterns of CG methylation and gene expression (Figs 3, 4). The double mutant classes showed variability in the degree of CG methylation loss and changes to gene expression, with the aabbDD mutants showing the greatest change compared with the AAbbdd and aaBBdd double mutants (Figs 3, 4). This suggests that *MET1-D1* is less effective at compensating for the loss of the other two homoeologues than *MET1-A1* or *MET1-B1*. Furthermore, the double mutants did not show any growth defects, despite the aabbDD mutants having a similar degree of demethylation to the Aabbdd mutant (74.0% CG methylation and 72.4% CG methylation, respectively). Together these features suggest that double mutants with four mutant alleles may be a suitable compromise, allowing generation of plants with reduced CG methylation while minimizing growth defects (Fig. 6).

We observed novel, heritable phenotypic variation in one of the segregating *MET1-1* populations (Fig. 5). These phenotypes (delayed flowering and the development of awns) were both identified in plants that had four out of six non-functional copies of *MET1-1*, indicating that a null *met1-1* mutant is not necessary to generate phenotypic variation. Loss of DNA methylation can generate novel variation in multiple ways. Firstly, epialleles may be generated, resulting in altered patterns of gene expression. Secondly, hypomethylation of TEs is associated with their transcription and remobilization, leading to abnormal developmental phenotypes (Miura *et al.*, 2001; Mirouze *et al.*, 2009; Reinders *et al.*, 2009; Tsukahara *et al.*, 2009). We found that one fifth of TEs were associated with at least one DMR in the Aabbdd mutant, and 45 of these TEs had up-regulated expression, potentially resulting in their activation. This may explain the stunted growth of the Aabbdd mutant, and the severely reduced viability of higher order *met1-1* mutants. Genome instability caused by loss of DNA methylation can also cause structural variation (Zhang *et al.*, 2023). We demonstrated that the spontaneous awned *met1-1* mutant had a substantial deletion of *B1*, however we were unable to determine if this was caused by changes in CG methylation (Supplementary Fig. S18). A similar large deletion was found in wheat treated with the demethylation agent zebularine; the deletion encompassed *FT-B1* and was associated with an increase in spikelet number and a delay in flowering (Finnegan *et al.*, 2019). Further work is required to determine whether the observed late flowering phenotype and the deletion of *B1* were caused by loss of CG methylation or unrelated genetic changes.

### Lethal effect of null *met1-1* mutants associated with mutant (abd) gametes

We observed a clear segregation distortion when genotyping selfed seed from a triple heterozygous (AaBbDd) *MET1-1* parent, with under-representation of higher level mutants (5/6 and 6/6 non-functional copies). We attributed this distortion to a reduced transmission of gametes with no functional copy of *MET1-1* (abd gametes) when donated maternally or paternally. In reciprocal crosses, abd gametes transmitted paternally were rarer than abd gametes transmitted maternally, whereas the opposite trend was observed using selfed heterozygous (AaBbDd) mutants. This discrepancy may be explained by post-fertilization effects of *met1-1* being made more severe in a scenario where mutant alleles may be present in both gametes, as for the selfed heterozygous plants. This is consistent with work in Arabidopsis that found that selfed heterozygous *met1* mutants had higher rates of embryo abortion than WT×homozygous *met1* mutant reciprocal crosses (Xiao *et al.*, 2006). The reduced maternal transmission of the mutant *MET1-1* alleles in the selfed plants could be explained by the three mitotic divisions that occur in female gametogenesis after meiosis, compared with only two post-meiotic divisions in male gametogenesis (Liu and Qu, 2008). As MET1 is responsible for maintaining CG methylation following DNA replication (Zhang *et al.*, 2018), a greater depletion in CG methylation would occur in female gametes lacking a functional copy of *MET1-1* compared with male gametes.

In Arabidopsis, transmission of mutant *met1* alleles has also been shown to be distorted in the gametes. However, in contrast to our results where both maternal and paternal transmission was affected, only the paternal transmission of *met1* was reduced to 36.8% of the expected rate in Arabidopsis, while maternal transmission was not reduced (Liang *et al.*, 2022). This single parental bias in *met1* transmission was, in part, due to impaired pollen development resulting in abnormal numbers of nuclei in the pollen grains. In our work, we found no change to any pollen phenotype measured in the *met1-1* mutant plants compared with WT segregant plants, suggesting that instead the barrier to the production of null mutants may occur during fertilization and/or embryogenesis. Post-zygotic effects are also likely to be important since we found >9 times more offspring with five non-functional *MET1* alleles in grains than in leaves of seedlings. Reduced germination may be due to abnormal endosperm development as observed in Arabidopsis *met1* mutants (Jullien *et al.*, 2006). This would also be consistent with the smaller seed sizes we observed in wheat mutants inheriting *met1-1* alleles through abd gametes, although the effect was not as extreme as observed in rice (Hu *et al.*, 2014; Yamauchi *et al.*, 2014), nor was it exclusively paternal as in Arabidopsis (Xiao *et al.*, 2006).

### Reduced function of *MET1-1* is likely to be buffered by activation of other epigenetic pathways

Loss of five out of six functional copies of *MET1-1* resulted in a significant decrease in CG methylation, but an increase in CHG and CHH methylation (Fig. 3), suggesting that other methylation pathways are activated upon reduction of *MET1-1* activity. A similar increase in CHG and CHH methylation was observed in tomato *met1* mutants (Yang *et al.*, 2019) and in rice *MET1/met1* heterozygotes, but not in the homozygous *met1* mutant (Hu *et al.*, 2014). In Arabidopsis, CHG methylation was increased in *met1* mutants, while CHH methylation was decreased (Srikant *et al.*, 2022). In wheat, the increases in CHG and CHH methylation are found in extremely well-defined regions where CG methylation is maintained to at least the level of the AAbbdd double mutant (Fig. 3; Supplementary Figs S6–S12). Future studies are required to determine whether these regions feature only in species with large genomes, making them undetectable until now.

The compensatory increase in CHG and CHH methylation we observed may be promoted by transcriptional up-regulation of RdDM. We found increased expression of genes involved in generating siRNAs which direct the RdDM machinery to loci to be methylated (He *et al.*, 2009; Erdmann and Picard, 2020); these include RNA Polymerase IV/V Subunit 4 and the HEN1 RNA methyltransferase in the double and Aabbdd *met1-1* mutants (Fig. 4C). We also found down-regulation of a DME-like DNA glycosylase in the mutants with the largest reduction in CG methylation (aabbDD and Aabbdd) (Fig. 4C). Down-regulation of DNA glycosylases has also been observed in *met1* mutants in Arabidopsis and rice (Mathieu *et al.*, 2007; Hu *et al.*, 2014). Transcriptional down-regulation of the Arabidopsis *ROS1* DNA glycosylase is caused by loss of methylation in its promoter at the methylation monitoring sequence (MEMS) (Lei *et al.*, 2015). We found that the DME-like DNA glycosylase down-regulated in the aabbDD and Aabbdd *met1-1* mutants overlapped a hypo-DMR, suggesting that a similar MEMS mechanism may regulate the expression of this DNA glycosylase, allowing the plant to sense changes in the DNA methylation status and compensate for them.

Compensation for reduced CG methylation can also occur through changes in histone modifications. We observed transcriptional up-regulation of a SUVR4/SUVR1/SUVR2 histone lysine methyltransferase in the double and Aabbdd *met1-1* mutants (Fig. 4C). SUVR4/SUVR1/SUVR2 histone lysine methyltransferases have a preference for H3K9 methylation (Thorstensen *et al.*, 2006), suggesting that this epigenetic mark may also be altered upon reduction of CG methylation in wheat, as has been reported in Arabidopsis *met1* mutants (Tariq *et al.*, 2003; Mathieu *et al.*, 2007).

In summary, we leveraged partial redundancy between homoeologues in polyploid wheat to recover *met1-1* mutants. Similar to other plants with large genomes, complete null mutants were lethal. However, wheat plants with four null alleles (i.e. retaining two functional alleles) develop normally, have substantially reduced CG methylation, and occasionally exhibit novel heritable phenotypes. Generating partial mutants may also be effective in other polyploid species to manipulate CG methylation without lethal consequences, opening up new avenues to understand the role of CG methylation in polyploid species.

## Supplementary data

The following supplementary data are available at *JXB* online.

Fig. S1. Expression profiles of Ta*MET1* genes in the cultivar Azhurnaya.

Fig. S2. Overview of the tetraploid *met1-1* TILLING mutants and how they were used to generate mutant populations.

Fig. S3. The proportion of grains in each width category, for grains with a total of three or four mutant *met1-1* alleles.

Fig. S4. Percentage of cytosine methylation across genes and transposable elements.

Fig. S5. Absolute difference in percentage methylation between the WT segregant (AABBDD) and the AAbbDD mutant, the AAbbdd mutant, and the Aabbdd mutant across chromosome 5D.

Fig. S6. Percentage of methylated cytosines across the group 1 chromosomes for the AABBDD genotype and the AAbbDD, Aabbdd, and Aabbdd mutants.

Fig. S7. Percentage of methylated cytosines across the group 2 chromosomes for the AABBDD genotype and the AAbbDD, Aabbdd, and Aabbdd mutants.

Fig. S8. Percentage of methylated cytosines across the group 3 chromosomes for the AABBDD genotype and the AAbbDD, Aabbdd, and Aabbdd mutants.

Fig. S9. Percentage of methylated cytosines across the group 4 chromosomes for the AABBDD genotype and the AAbbDD, Aabbdd, and Aabbdd mutants.

Fig. S10. Percentage of methylated cytosines across the group 5 chromosomes for the AABBDD genotype and the AAbbDD, Aabbdd, and Aabbdd mutants.

Fig. S11. Percentage of methylated cytosines across the group 6 chromosomes for the AABBDD genotype and the AAbbDD, Aabbdd, and Aabbdd mutants.

Fig. S12. Percentage of methylated cytosines across the group 7 chromosomes for the AABBDD genotype and the AAbbDD, Aabbdd, and Aabbdd mutants.

Fig. S13. Percentage of methylated cytosines across chromosome 5D for each of the single and double mutants.

Fig. S14. Number of hypermethylated differentially methylated regions (hyper-DMRs) relative to AABBDD in each genotype.

Fig. S15. Number of hypomethylated and hypermethylated DMRs per kb in each of the chromosome regions.

Fig. S16. The percentage of differentially expressed genes (DEGs) and differentially expressed transposons (DETs) associated with a hypo-DMR (differentially methylated region).

Fig. S17. The late flowering phenotype in the C0451×C1292×C2028 *MET1-1* population was independent of *MET1-1* genotype and was accompanied by increased plant height and number of fully formed spikelets.

Fig. S18. The observed awned phenotype in the C0451×C1292×C2028 *MET1-1* population followed a single gene semi-dominant Mendelian inheritance pattern, which was caused by a deletion encompassing *Tipped1* (*B1*).

Table S1. Primer sequences used for KASP genotyping and PCR.

Table S2. Segregation distortion of *met1-1* genotypes by mutant copy number in the C0451×C2028×C1292 population F_2_ generation.

Table S3. Grain genotyping results validated by leaf genotyping for plants with three marker results.

Table S4. Segregation distortion of Kronos F_4_ grain genotypes by mutant copy number in selfed double heterozygous (AaBb) plant offspring.

Table S5. Pollen phenotypes are not significantly different between *met1-1* mutants and AABBDD plants.

Table S6. Transposable elements (TEs) that intersect at least one differentially methylated region (DMR).

eraf016_suppl_Supplementary_Material

## Data Availability

Raw reads from whole-genome bisulfite sequencing have been deposited in the European Nucleotide Archive under project PRJEB77426, and reads from RNA-sequencing have been deposited under project PRJEB77425. All scripts used for whole-genome bisulfite sequencing and RNA-sequencing analyses can be found on GitHub at https://github.com/Borrill-Lab/Wheat_MET1/.
