# Supplementary material for: Partial redundancy buffers deleterious effects of mutating DNA methyltransferase 1-1 (MET1-1) in polyploid wheat
Source: J Exp Bot. 2025 Apr 7;76(9):2500–16. doi: 10.1093/jxb/eraf016 (PMC12192439; doi:10.1093/jxb/eraf016)
Supplement: eraf016_suppl_Supplementary_Material [file eraf016_suppl_supplementary_material.pdf]

Supplementary Figures and Tables for "Partial redundancy buffers deleterious effects of mutating *DNA methyltransferase 1-1* (*MET1-1*) in polyploid wheat"

Samuel Burrows[1]*, Delfi Dorussen[1]*, Joseph Crudgington[1], Giorgia Di Santolo[1], James Simmonds[1], Marco Catoni[2], Philippa Borrill[1†]

[1] Department of Crop Genetics, John Innes Centre, Norwich Research Park, Norwich, NR4 7UH, United Kingdom.

[2] School of Biosciences, University of Birmingham, Birmingham, B15 2TT, United Kingdom.

* These authors contributed equally

[†] Corresponding author, email: philippa.borrill@jic.ac.uk

Supplementary Figures

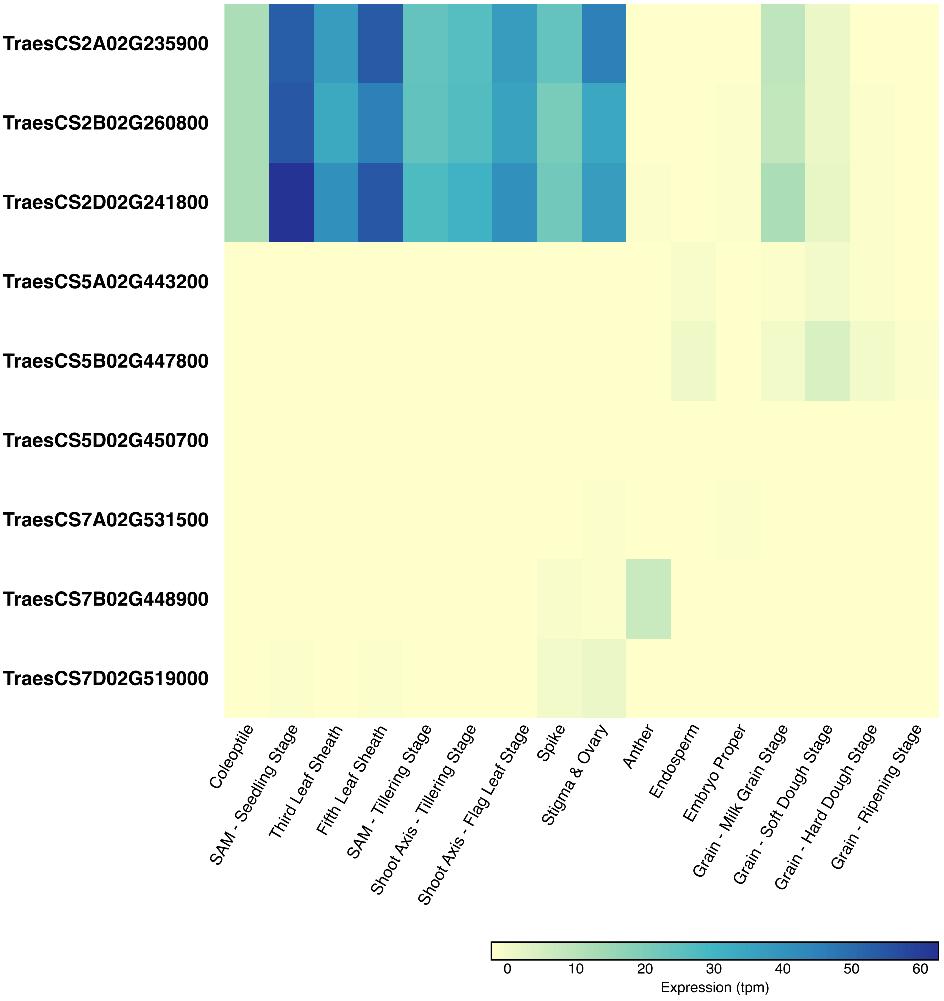

Supplementary Figure S1. Expression profiles of Ta*MET1* genes in the cultivar Azhurnaya showing that the chromosome 2 homoeologue group formed of *TraesCS2A02G 235900* , *TraesCS2B02G 260800* and *TraesCSD02G 241800* is the most highly expressed compared to homoeologue groups on chromosome 5 (*TraesCS5A02G 443200* , *TraesCS5B02G 447800* , *TraesCS5D02G 450700*) and chromosome 7 (*TraesCS7A02G 531500* , *TraesCS7B02G 448900* , *TraesCS7D02G 519000*). Data from Ramírez-González et al. (2018).

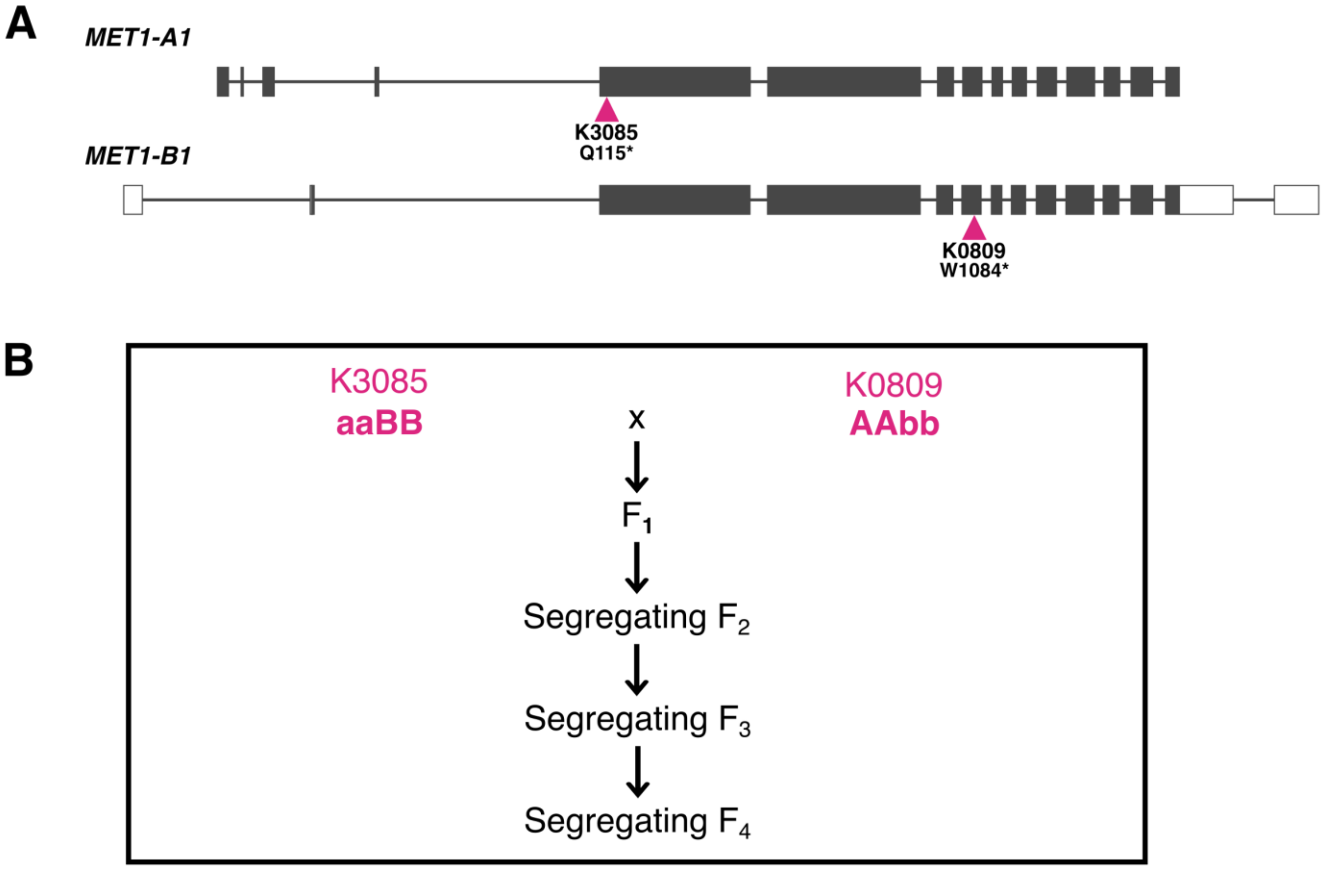

Supplementary Figure S2. Overview of the tetraploid *met1-1* TILLING mutants and how they were used to generate mutant populations. A) Structure of the two *MET1-1* homoeologues. Filled rectangles represent exons, empty rectangles represent untranslated regions, and lines represent introns. Triangles indicate the positions of the PTC mutations in the TILLING lines used to produce the segregating populations. B) Crossing structure showing the formation of the segregating populations in the F_2, F_3 and F_4 generations.

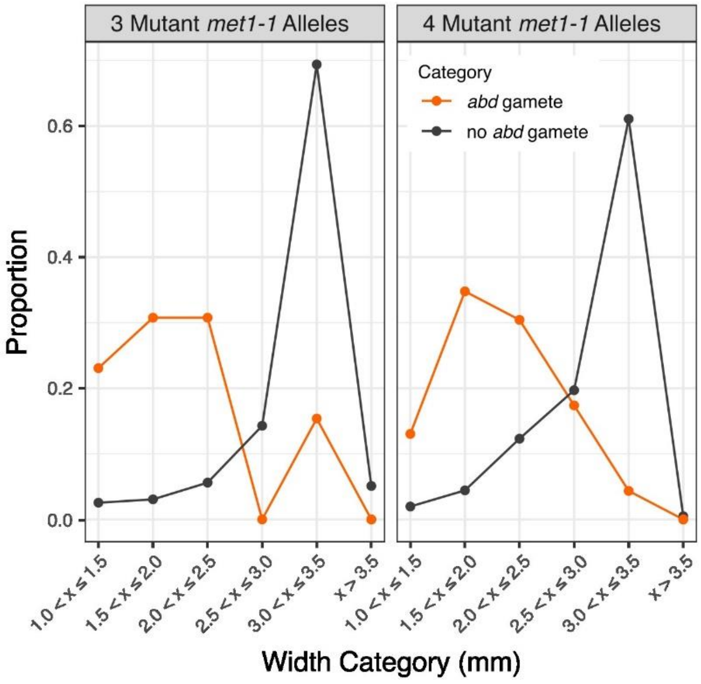

Supplementary Figure S3. The proportion of grains in each width category, for grains with a total of three or four mutant *met1-1* alleles, formed from an abd gamete (orange) or not (grey).

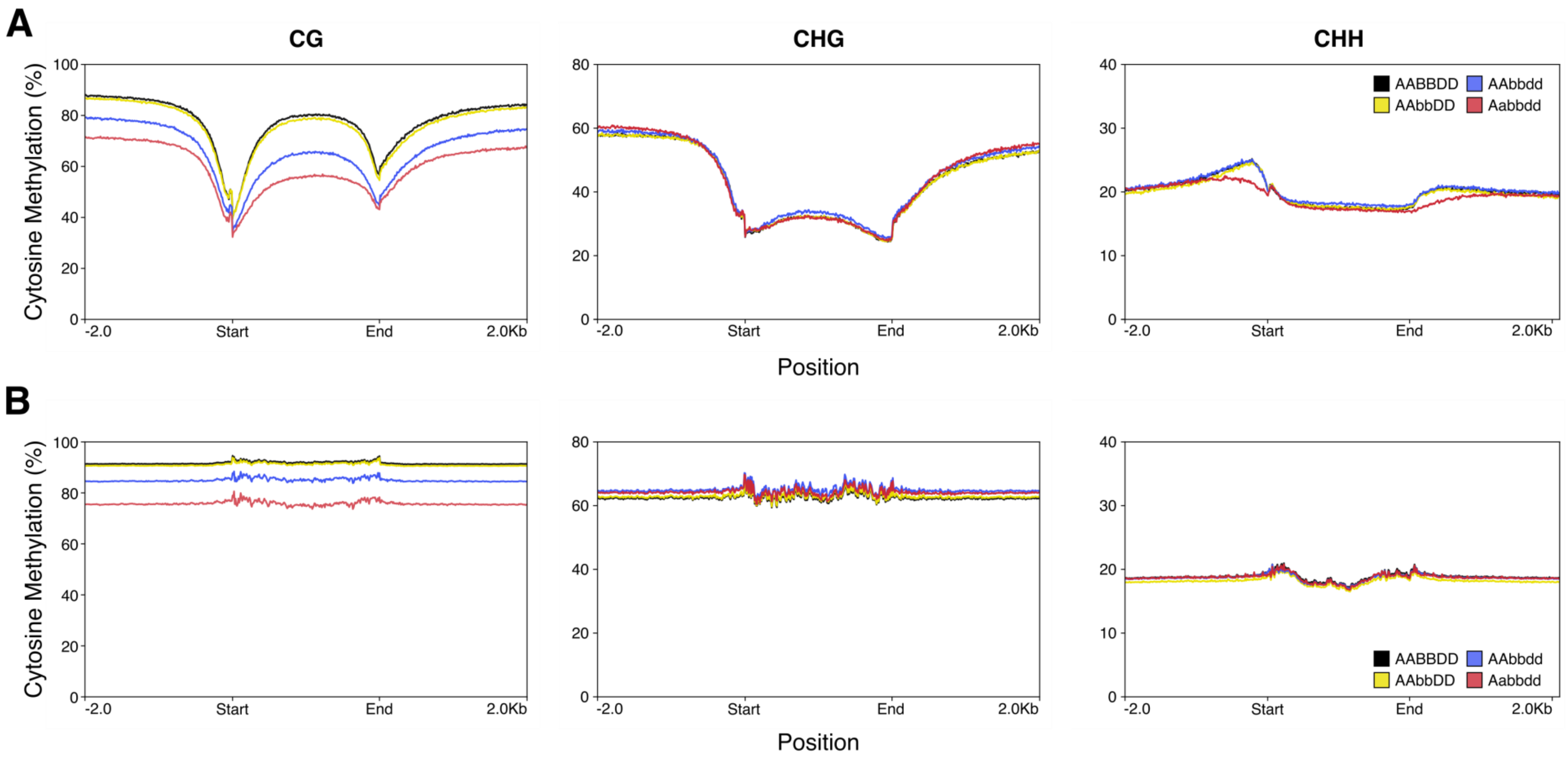

Supplementary Figure S4. Percentage cytosine methylation across genes (A) and transposable elements (B), including 2 kb up and downstream of the feature. Cytosine methylation in the CG (left), CHG (centre), and CHH (right) contexts are shown. The WT segregant (AABBDD, black), AAbbDD single mutant (yellow), AAbbdd double mutant (blue), and Aabbdd mutant (red) are shown.

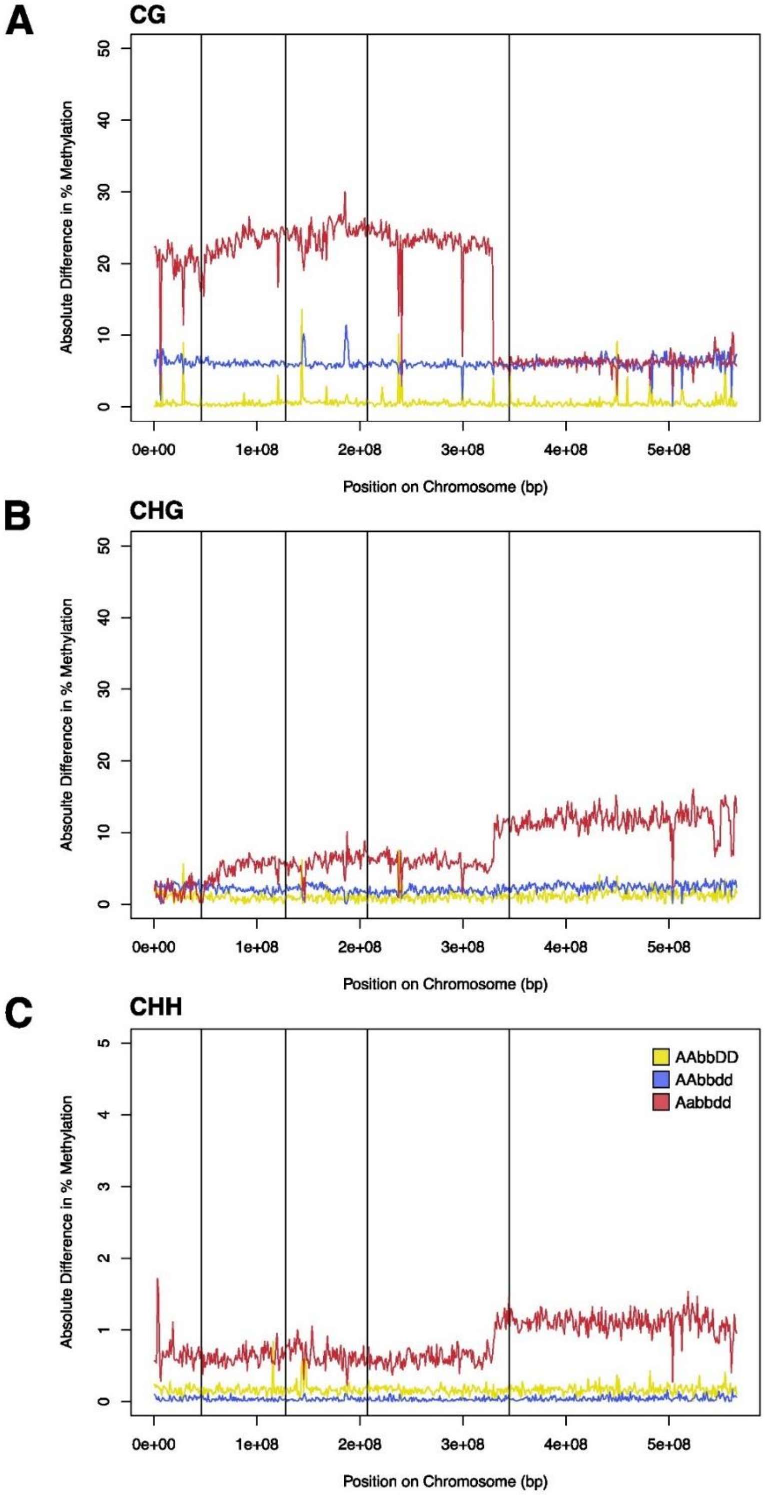

Supplementary Figure S5. Absolute difference in percentage methylation between the WT segregant (AABBDD) and the AAbbDD mutant (yellow), the AAbbdd mutant (blue) and the Aabbdd mutant (red) across Chromosome 5D, calculated across 1 Mb bins. Methylation in the CG (A), CHG (B), and CHH (C) contexts are shown. Vertical lines represent the boundaries between chromosomal regions R1 (leftmost), R2a, C, R2b and R3 (rightmost).

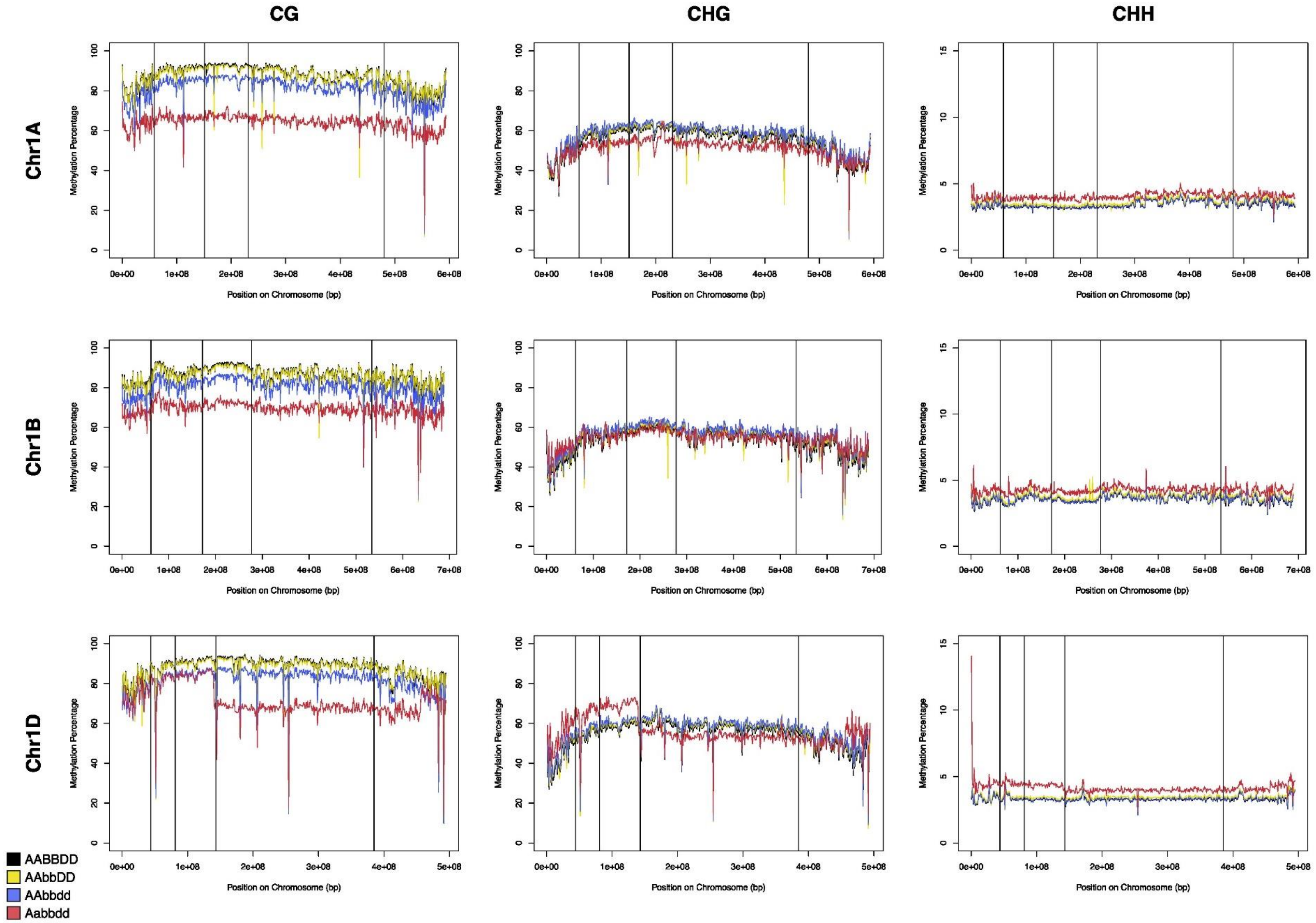

Supplementary Figure S6. Percentage methylated cytosines (in the CG, CHG and CHH contexts) calculated for 1 Mb bins across the group 1 chromosomes for the AABBDD genotype (black) and the AAbbDD (yellow), AAbbdd (blue) and Aabbdd (red) mutants. Vertical lines represent the boundaries between chromosomal regions R1 (leftmost), R2a, C, R2b and R3 (rightmost).

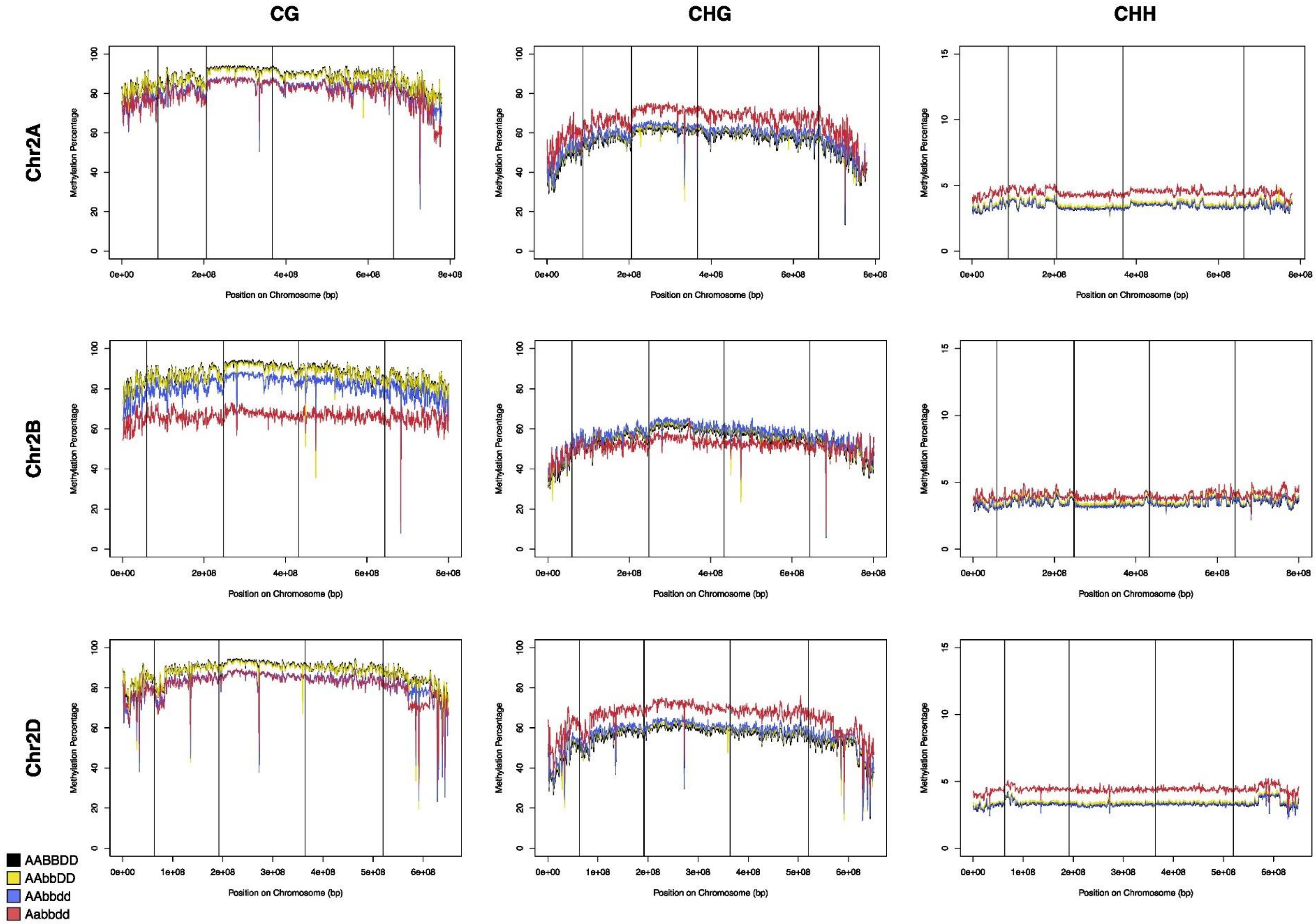

Supplementary Figure S7. Percentage methylated cytosines (in the CG, CHG and CHH contexts) calculated for 1 Mb bins across the group 2 chromosomes for the AABBDD genotype (black) and the AAbbDD (yellow), AAbbdd (blue) and Aabbdd (red) mutants. Vertical lines represent the boundaries between chromosomal regions R1 (leftmost), R2a, C, R2b and R3 (rightmost).

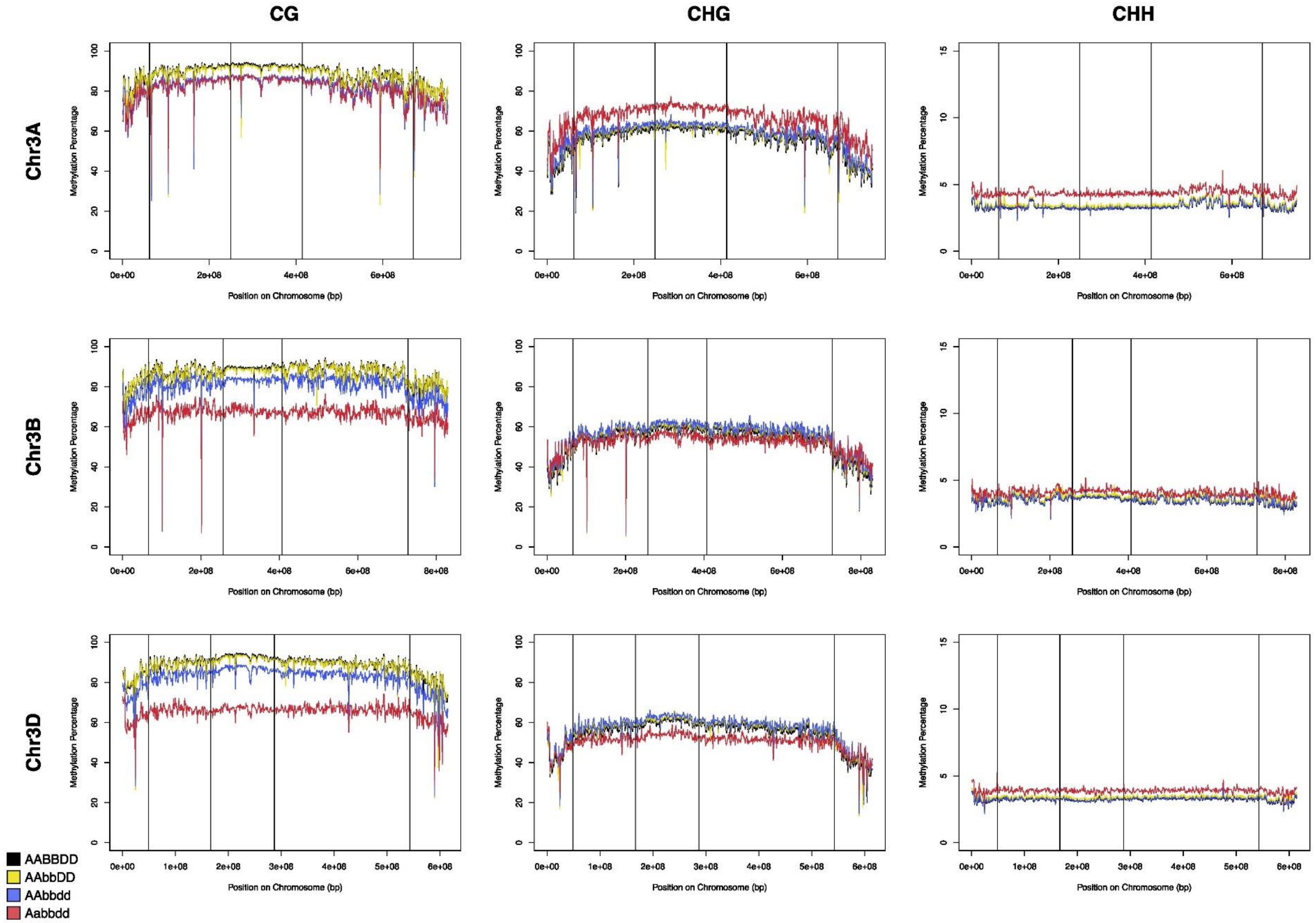

Supplementary Figure S8. Percentage methylated cytosines (in the CG, CHG and CHH contexts) calculated for 1 Mb bins across the group 3 chromosomes for the AABBDD genotype (black) and the AAbbDD (yellow), AAbbdd (blue) and Aabbdd (red) mutants. Vertical lines represent the boundaries between chromosomal regions R1 (leftmost), R2a, C, R2b and R3 (rightmost).

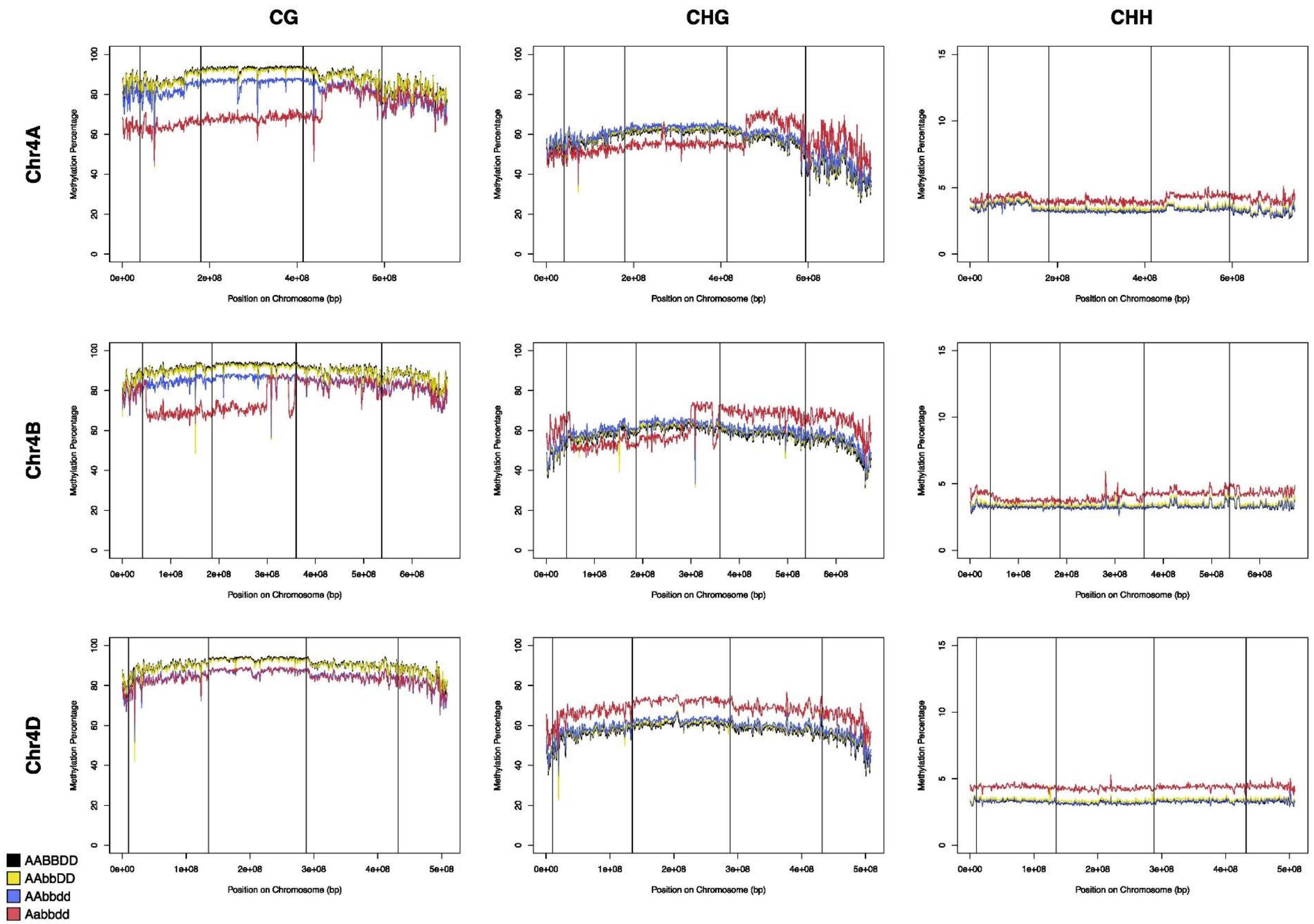

Supplementary Figure S9. Percentage methylated cytosines (in the CG, CHG and CHH contexts) calculate for 1 Mb bins across the group 4 chromosomes for the AABBDD genotype (black) and the AAbbDD (yellow), AAbbdd (blue) and Aabbdd (red) mutants. Vertical lines represent the boundaries between chromosomal regions R1 (leftmost), R2a, C, R2b and R3 (rightmost).

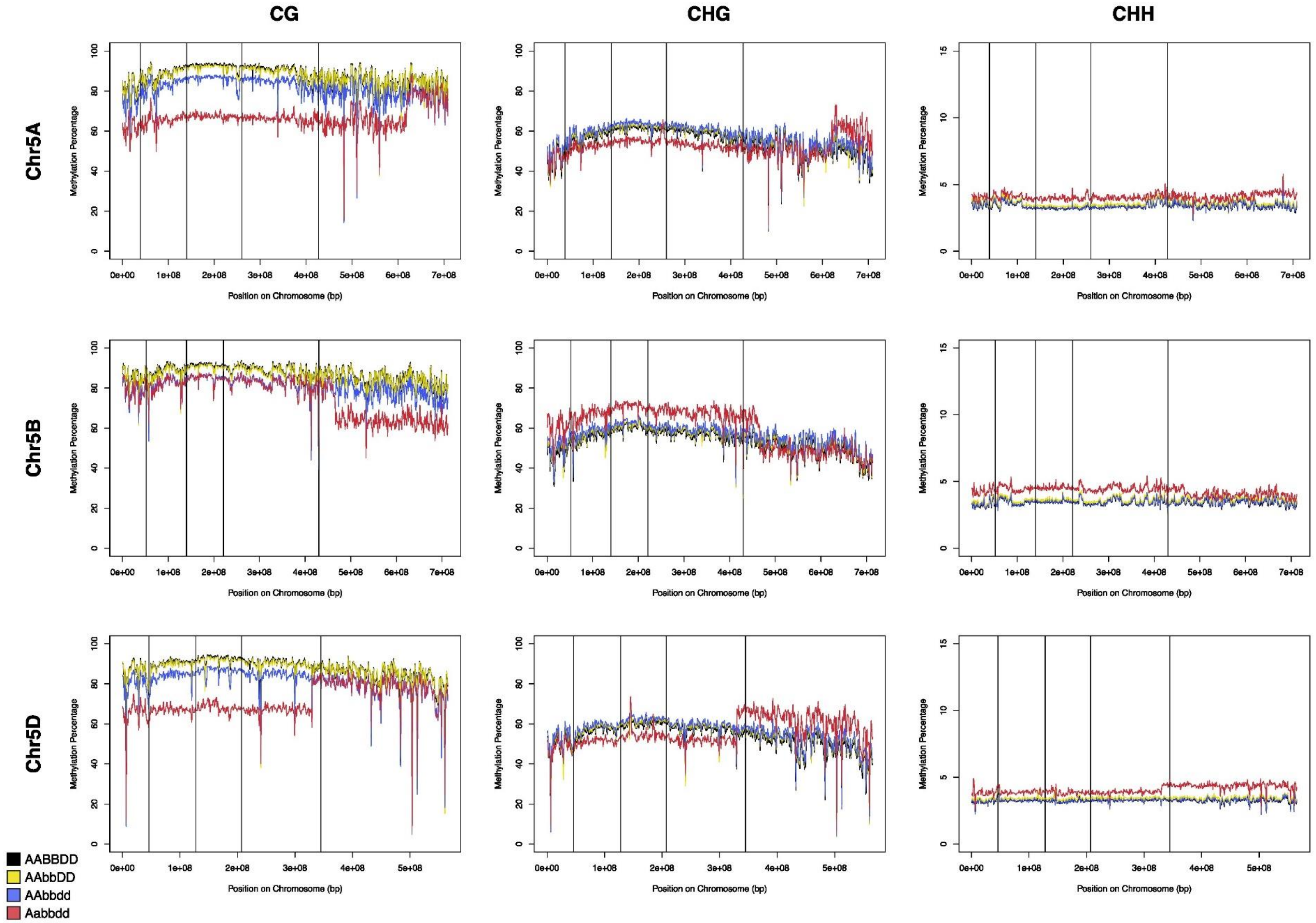

Supplementary Figure S10. Percentage methylated cytosines (in the CG, CHG and CHH contexts) calculated for 1 Mb bins across the group 5 chromosomes for the AABBDD genotype (black) and the AAbbDD (yellow), AAbbdd (blue) and Aabbdd (red) mutants. Vertical lines represent the boundaries between chromosomal regions R1 (leftmost), R2a, C, R2b and R3 (rightmost).

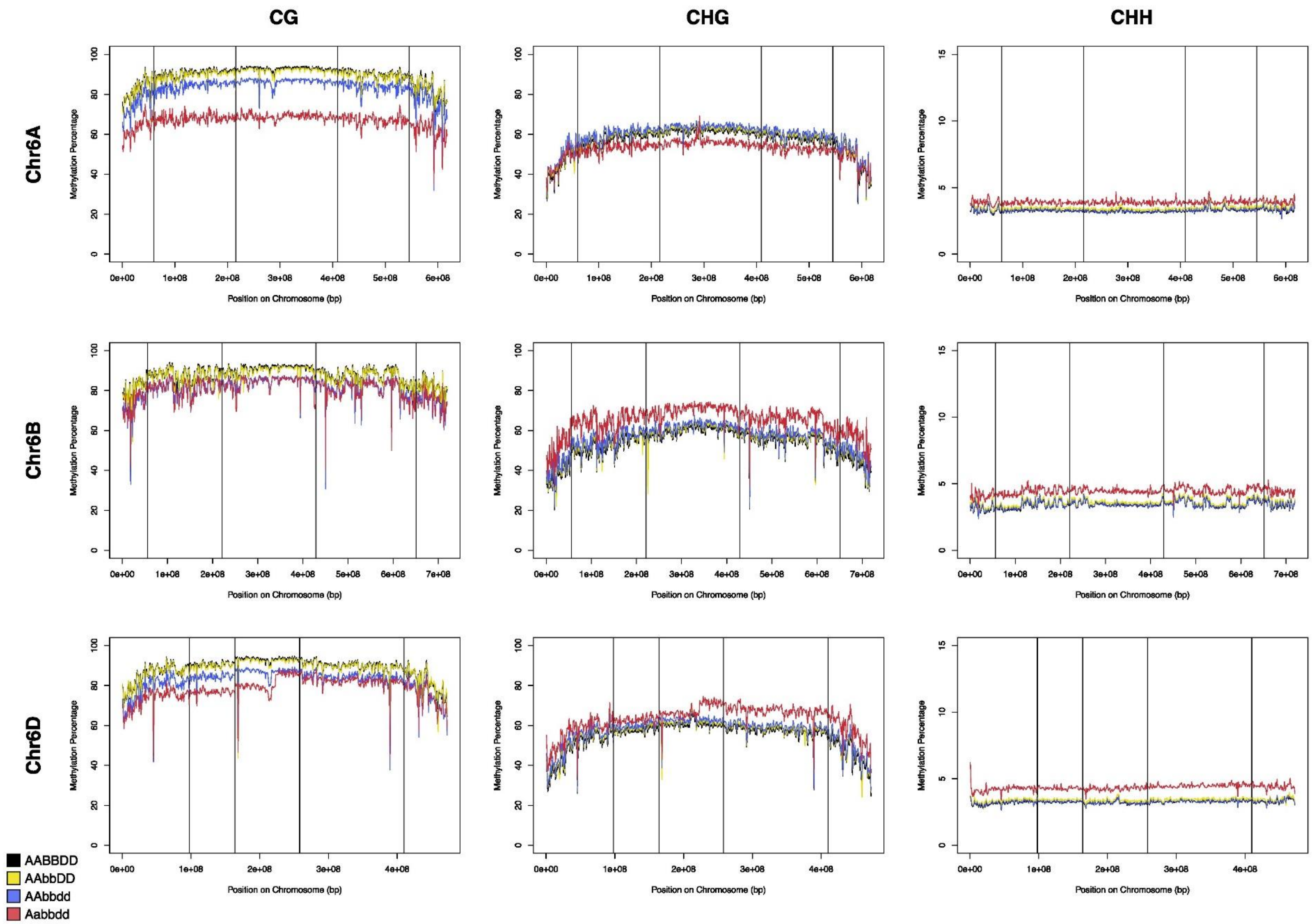

Supplementary Figure S11. Percentage methylated cytosines (in the CG, CHG and CHH contexts) calculated for 1 Mb bins across the group 6 chromosomes for the AABBDD genotype (black) and the AAbbDD (yellow), AAbbdd (blue) and Aabbdd (red) mutants. Vertical lines represent the boundaries between chromosomal regions R1 (leftmost), R2a, C, R2b and R3 (rightmost).

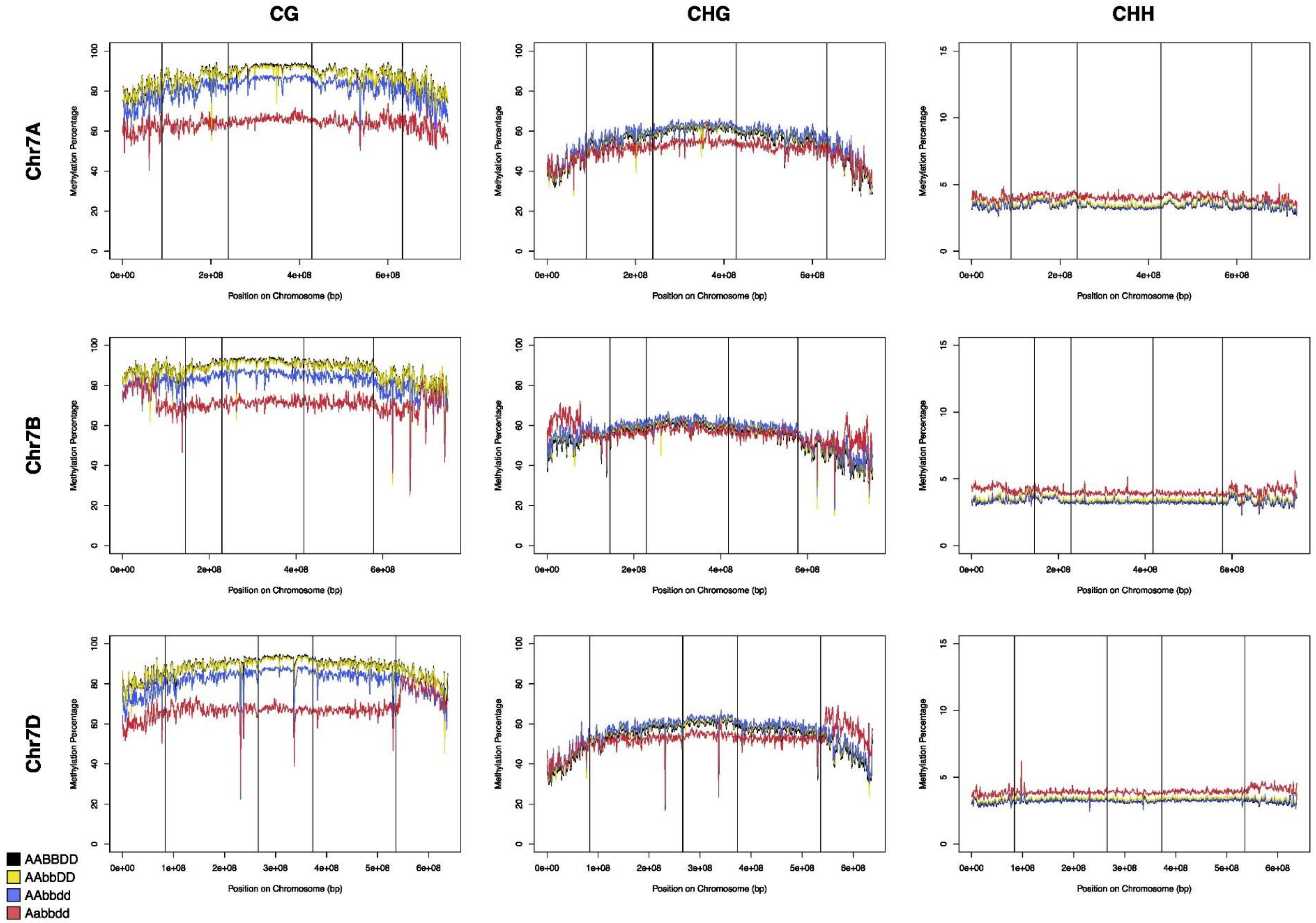

Supplementary Figure S12. Percentage methylated cytosines (in the CG, CHG and CHH contexts) calculated for 1 Mb bins across the group 7 chromosomes for the AABBDD genotype (black) and the AAbbDD (yellow), AAbbdd (blue) and Aabbdd (red) mutants. Vertical lines represent the boundaries between chromosomal regions R1 (leftmost), R2a, C, R2b and R3 (rightmost).

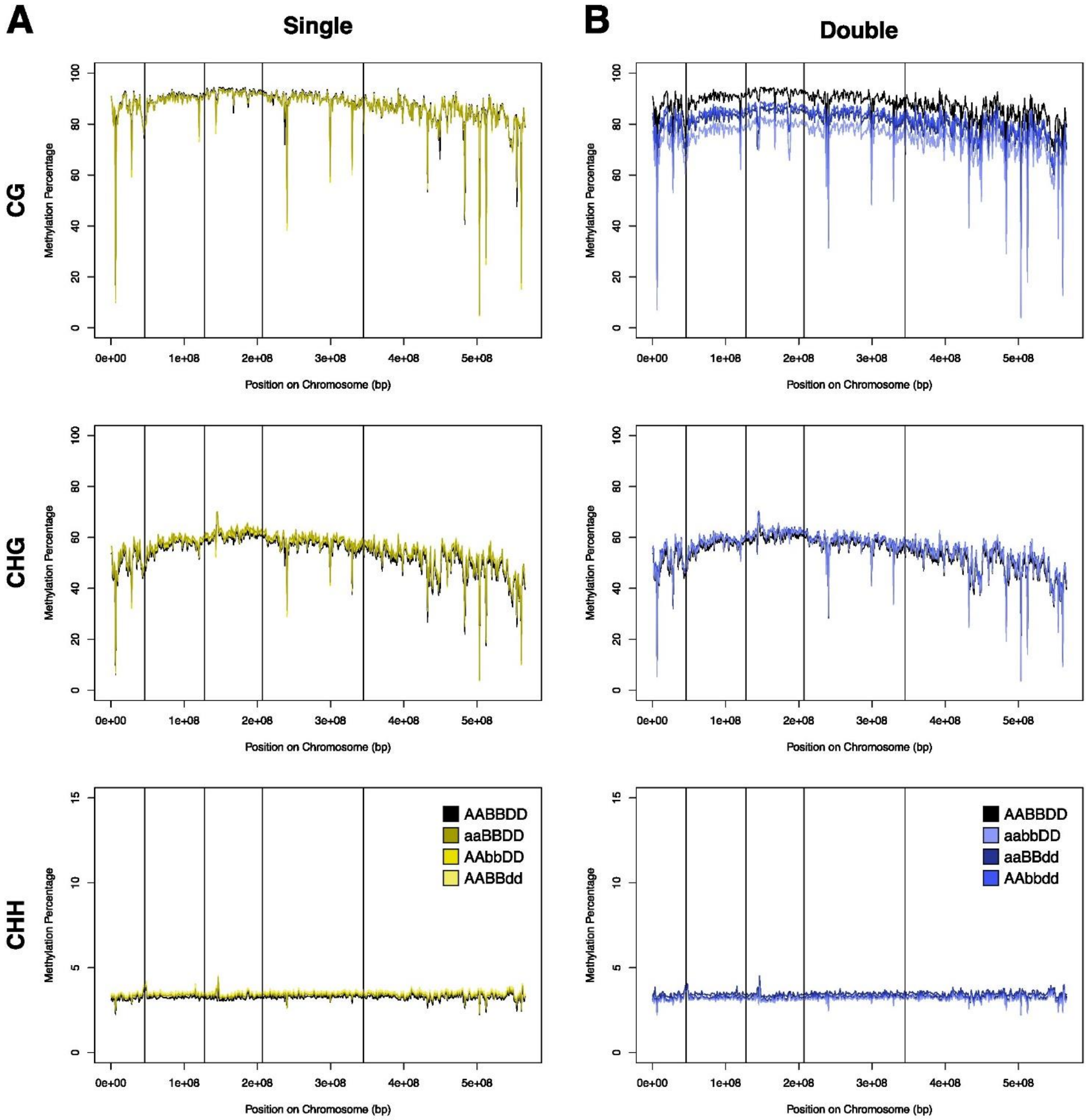

Supplementary Figure S13. Percentage of methylated cytosines in the CG, CHG, and CHH contexts across Chromosome 5D for each of the single mutants (A) and double mutants (B), calculated as an average in 1 Mb bins across the chromosome. Vertical lines represent the boundaries between chromosomal regions R1 (leftmost), R2a, C, R2b and R3 (rightmost).

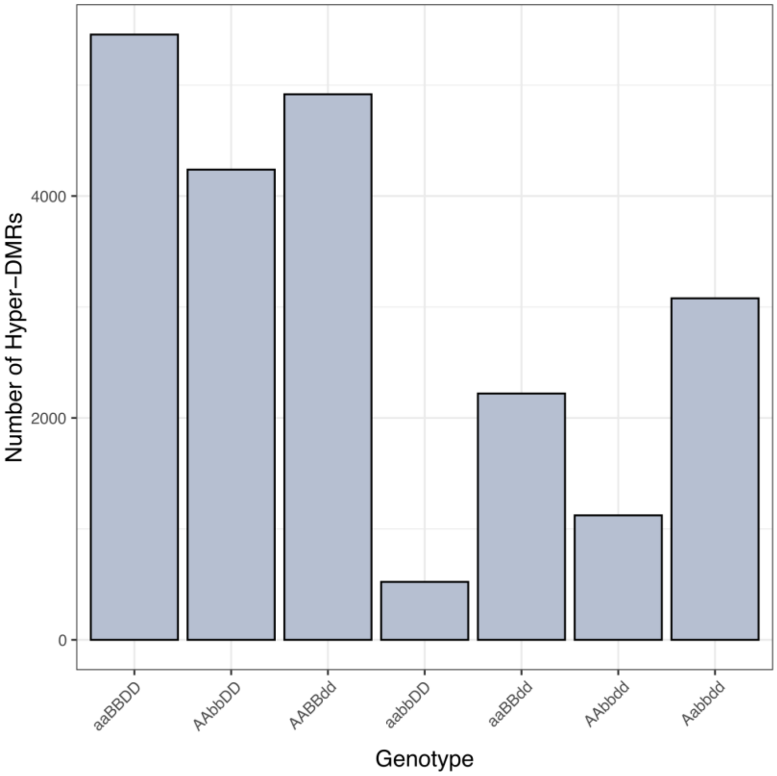

Supplementary Figure S14. Number of hyper-methylated differentially methylated regions (hyper-DMRs) relative to AABBDD in each genotype.

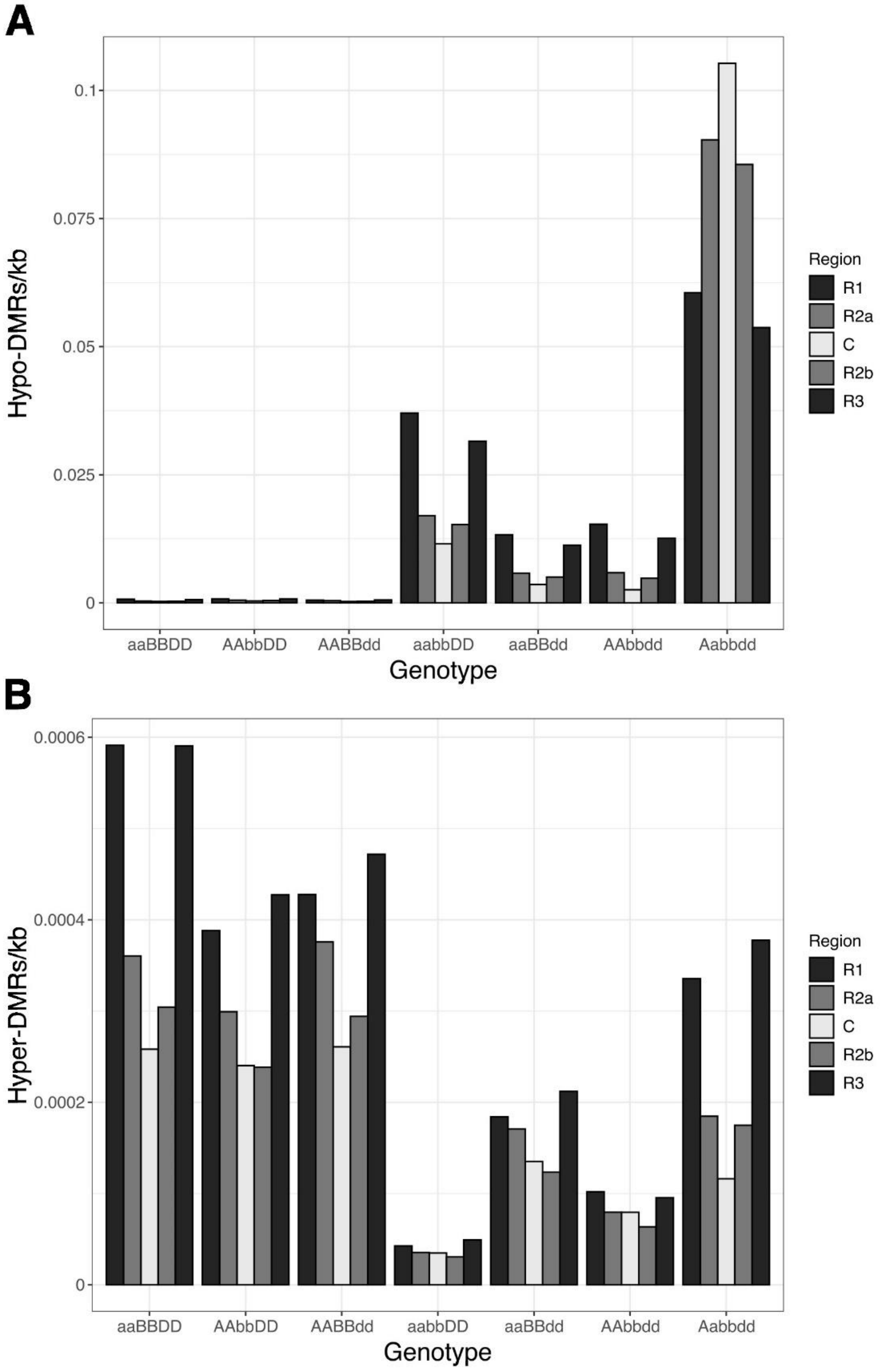

Supplementary Figure S15. Number of hypo-methylated (A) and hyper-methylated (B) DMRs per kb in each of the chromosome regions – the distal regions R1 and R3 are shown in dark grey, the proximal regions R2a and R2b in medium grey, and the centromeric region C in light grey.

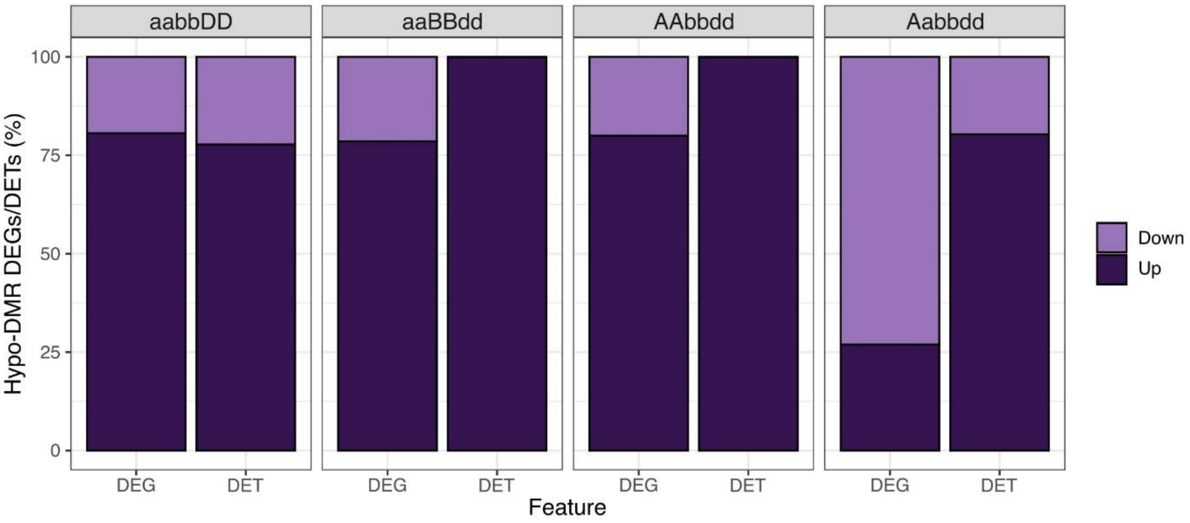

Supplementary Figure S16. The percentage of differentially expressed genes (DEGs) and differentially expressed transposons (DETs) associated with a hypo-DMR (differentially methylated region) which are down-regulated (light purple) or up-regulated (dark purple) in the double and Aabbdd *met1-1* mutants.

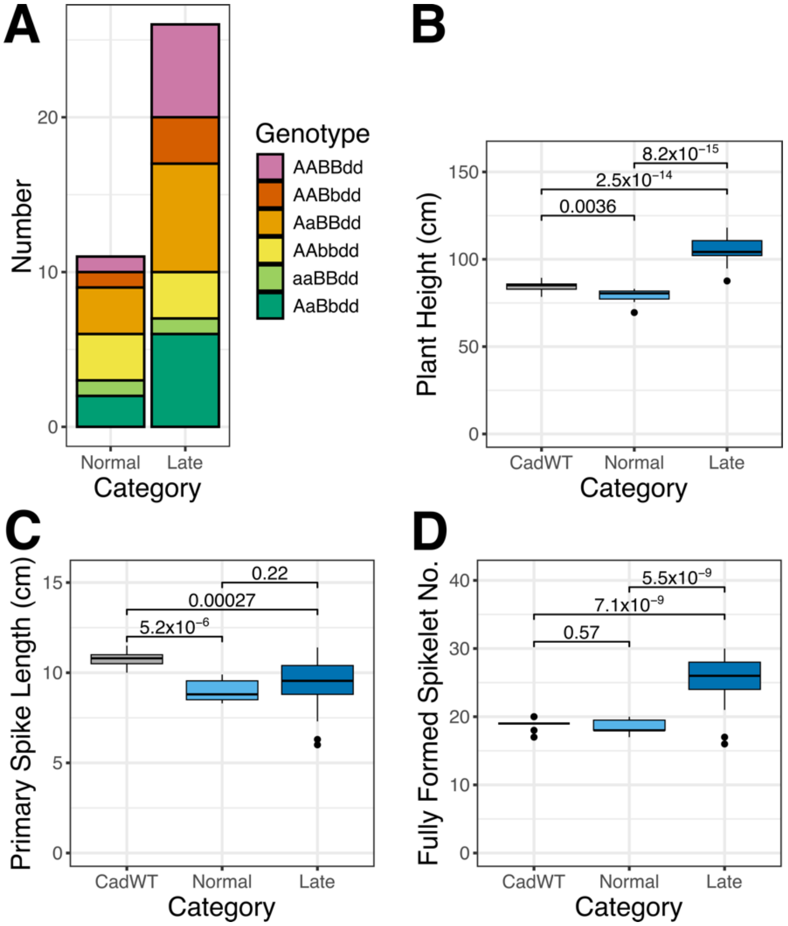

Supplementary Figure S17. The late flowering phenotype in the C0451xC1292xC2028 *MET1-1* population was independent of *MET1-1* genotype and was accompanied by increased plant height and number of fully formed spikelets. A) *MET1-1* genotypes of late and normal flowering plants. Plant height (B), primary spike length (C) and number of fully formed spikelets (D) for each of the phenotype categories. For (B) to (D), the means were compared by a t-test and the False Discovery Rate (FDR) adjusted p-value calculated is shown.

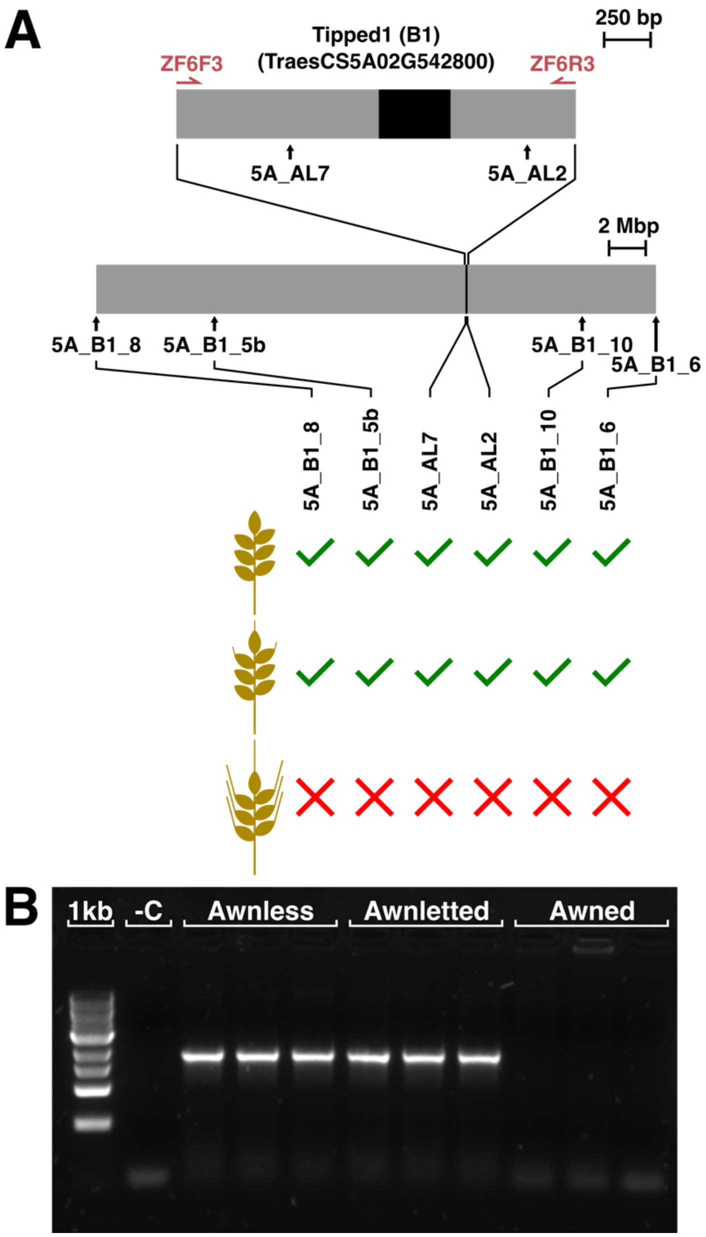

Supplementary Figure S18. The observed awned phenotype in the C0451xC1292xC2028 *MET1-1* population followed a single gene semi dominant mendelian inheritance pattern, which was caused by a deletion encompassing *Tipped1* (*B1*). A) Diagram of the *B1* gene (black) and surrounding region (grey) with KASP marker binding sites (black arrows) and PCR primer binding sites (maroon half arrows). Schematic summarising successful (green tick) and unsuccessful (red cross) amplification of KASP marker sets for each phenotype class of plant (awned, awnletted and awnless). B) Agarose gel image showing PCR product of the ZF6F3 and ZF6R3 primer set (expected product ≈2kb) from 3 individual F2 segregant plants of each phenotype class and 1kb DNA ladder (1kb) and no DNA (-C) controls.

Supplementary Tables

Supplementary Table S1. Primer sequences used for KASP genotyping and PCR.

**Name**	**Sequence (5’-3’)**	**Purpose**
Met1_2A_Cad0465_WT	GAAGGTCGGAGTCAACGGATTaatgaagttgattcaggcaaatG	KASP genotyping Cadenza C0465 x C0884 *MET1-1* population
Met1_2A_Cad0465_Mut	GAAGGTGACCAAGTTCATGCTaatgaagttgattcaggcaaatA	
Met1_2A_Cad0465_Com	ACAGCCAGCAAAAATGTCG	
Met1_2B_Cad0465_WT	GAAGGTCGGAGTCAACGGATTcccatacagacccttccactG	
Met1_2B_Cad0465_Mut	GAAGGTGACCAAGTTCATGCTcccatacagacccttccactA	
Met1_2B_Cad0465_Com	GTGTTTGTGAGATGTTAATTCCCA	
Met1_2D_Cad0884_WT1	GAAGGTCGGAGTCAACGGATTaaatatattggaactcctacatcctG	
Met1_2D_Cad0884_Mut1	GAAGGTGACCAAGTTCATGCTaaatatattggaactcctacatcctA	
Met1_2D_Cad0884_Com1	TGTCTTTGGATCTGGTTTTATGAC	
Met1_2A_Cad0451_WT	GAAGGTCGGAGTCAACGGATTcccctctgtttcttcatcatcttC	KASP genotyping Cadenza C0451 x C2028 x C1292 *MET1-1* population
Met1_2A_Cad0451_Mut	GAAGGTGACCAAGTTCATGCTcccctctgtttcttcatcatcttT	
Met1_2A_Cad0451_Com	AGTGTAAAGAGGTAGCTGAGGAT	
Met1_2D_Cad2028_WT	GAAGGTCGGAGTCAACGGATTttgggcgtattgaggactgG	
Met1_2D_Cad2028_Mut	GAAGGTGACCAAGTTCATGCTttgggcgtattgaggactgA	
Met1_2D_Cad2028_Com	GTTCTGTTGAGAGCCAGACT	
Met1_2B_Cad1292_F	GCAACAAATGGTGTTGAAAAGT	CAPS genotyping Cadenza C0451 x C2028 x C1292 *MET1-1* population
Met1_2B_Cad1292_R	GCCTTCTCATAAAAGTGGTTGA	
MET1-A_K3085_WT_H	GAAGGTCGGAGTCAACGGATTaggtgcagagcaaagccaC	KASP genotyping Kronos *MET1-1* population
MET1-A_K3085_M_F	GAAGGTGACCAAGTTCATGCTaggtgcagagcaaagccaT	
MET1-A_K3085_C	ccaattttccttgtgcggtgA	
MET1-B_K0809_WT_H	GAAGGTCGGAGTCAACGGATTctctctttgggcacTtccatC	
MET1-B_K0809_M_F	GAAGGTGACCAAGTTCATGCTctctctttgggcacTtccatT	
MET1-B_K0809_C	tcatgtctgtggctcgcaag	

Supplementary Table S2. Segregation distortion of *met1-1* genotypes by mutant copy number in the C0451xC2028xC1292 population F_2 generation.

**Mutant copy No.**	**Observed Count**	**Expected Count**
0	15	5.13
1	34	30.75
2	98	76.88
3	116	102.50
4	65	76.88
5	0	30.75
6	0	5.13
**Total**	328	328
**Chi Squared p-value**	$5.05x10^{-12}$	

Supplementary Table S3. Grain genotyping results validated by leaf genotyping for plants with 3 marker results. Perfect match = genotyping results of all three homoeologs using grain DNA match the results from leaf DNA.

	Number of plants	Percent match
**A Match**	162	100.0%
**B Match**	159	98.1%
**D Match**	160	98.8%
**Perfect Match**	157	96.9%
**Total Leaves**	162	

Supplementary Table S4. Segregation distortion of Kronos F_4 grain genotypes by mutant copy number in selfed double heterozygous (AaBb) plant offspring. Mutant *met1-1* alleles were classified as paternally or maternally inherited by grain genotyping.

Mutant copy No.	Observed Count	Expected Count
0	44	20.75
1	135	83.00
2	142	124.50
3	11	83.00
4	0	20.75
**Total**	332	332
**Chi Squared p-value**	3.39×10^{-30}	

No. Paternal Mutant Copies	Observed Count	Expected Count
0	101	83
1	213	166
2	18	83
**Total**	332	332

No. Maternal Mutant Copies	Observed Count	Expected Count
0	130	83
1	201	166
2	1	83
**Total**	332	332

Supplementary Table S5. Pollen phenotypes are not significantly different between *met1-1* mutants and AABBDD plants. The estimated marginal means for each phenotype are shown for each genotype category. Shared letters show genotypes that are not significantly different from one another (FDR adjusted p<0.05).

**Genotype Category**	**Pollen Grains per Anther**	**Modal Pollen Diameter (µm)**	**Non-Viable Pollen Grains (%)**	**Non- and Mononucleated Pollen Grains (%)**
Cadenza WT	2,459 (a)	49.8 (a)	1.05 (a)	8.41 (a)
AABBDD	1,829 (bc)	46.2 (b)	2.62 (b)	12.45 (ab)
aaBBDD/AAbbDD/AABBdd	2,085 (ab)	46.8 (b)	1.96 (b)	-
aabbDD/aaBBdd/AAbbdd	1,623 (c)	45.3 (b)	1.60 (ab)	11.40 (a)
AaBbdd/AabbDd/aaBbDd	1,780 (bc)	45.8 (b)	2.10 (b)	19.56 (b)
N per genotype category	10-15	10-16	16-38	4-15

Supplementary Table S6. Transposable elements (TEs) that intersect at least one differentially methylated region (DMR), categorised according to class and family, as a percentage of the total number of TEs that overlap a DMR in each mutant genotype. Class I TEs include long terminal repeat (LTR) retrotransposons which consist largely of Copia and Gypsy elements. Class II TEs consist largely of terminal inverted repeat (TIR) transposons. TEs which are not Class I or Class II are unclassified.

			**aaBBDD**	**AAbbDD**	**AABBdd**	**aabbDD**	**aaBBdd**	**AAbbdd**	**Aabbdd**
**Class I**			**81.0**	**82.0**	**84.8**	**74.7**	**74.2**	**60.0**	**88.2**
	LTR		79.3	80.2	83.2	72.5	72.2	56.5	87.0
		Copia	16.7	15.6	16.9	18.4	17.3	12.5	20.5
		Gypsy	54.7	57.0	59.6	47.8	48.3	36.5	61.3
**Class II**			**13.5**	**13.6**	**11.8**	**18.5**	**18.7**	**28.9**	**9.5**
	TIR		13.5	13.6	11.5	18.4	18.6	28.6	9.3
**Unclassified**			**5.5**	**4.4**	**3.4**	**6.8**	**7.0**	**11.1**	**2.3**
